# A Systematic Review of Creativity-Related Studies Applying the Remote Associates Test From 2000 to 2019

**DOI:** 10.3389/fpsyg.2020.573432

**Published:** 2020-10-23

**Authors:** Ching-Lin Wu, Shih-Yuan Huang, Pei-Zhen Chen, Hsueh-Chih Chen

**Affiliations:** ^1^Program of Learning Sciences, National Taiwan Normal University, Taipei, Taiwan; ^2^Institute for Research Excellence in Learning Sciences, National Taiwan Normal University, Taipei, Taiwan; ^3^Department of Educational Psychology and Counseling, National Taiwan Normal University, Taipei, Taiwan; ^4^Chinese Language and Technology Center, National Taiwan Normal University, Taipei, Taiwan

**Keywords:** remote association, remote associates test, creativity, trend, review

## Abstract

The study examines how the remote associates test (RAT) has been used to examine theories of creativity through a review of recent studies on creativity. Creativity-related studies published between 2000 and 2019 were retrieved from the SCOPUS database. A total of 172 papers were chosen for further analysis. Content analysis shows that research on creativity using RAT mainly concerns remote association, insight problem-solving, general creative process, test development, individual difference, effect of treatment, clinical case, social interaction effect, and predictor or criterion. The study constructs a theoretical framework based on the 4P (Product–Person–Process–Place) model and demonstrates how empirical studies using the RAT explore the individual differences, internal processes, and external influences of creative thinking. In addition, the most commonly used version of the RAT is the Compound Remote Associates Problems (Bowden and Jung-Beeman, 2003a). Current research shows a trend whereby the creative thinking process has been receiving greater attention. In particular, a growing number of studies in this field have been carried out using cognitive neuroscience technologies. These findings suggest that the RAT provides researchers with a way to deepen their understanding of different levels of creativity.

## Introduction

Creativity is a complicated cognitive function; it has been given various definitions by scholars from diverse backgrounds specializing in different research orientations on creativity (Sternberg and Lubart, 1999). The 4Ps Model of Creativity (Rhodes, 1961) is often cited as a theoretical framework. It examines novel and appropriate products created by an individual, explores the personality of the creative person, analyzes the environment that enables people to produce creativity, and studies the process of how individuals generate creativity. Regardless of the research orientation, a tool that measures creativity with high reliability and validity is essential. The remote associates test (RAT) is a reliable tool that evaluates individuals' creative potential (Mednick, 1968). The creative process during remote associates problem solving involves two stages—an initial divergent stage of idea generation and then a convergent stage of solution matching and evaluation (Smith et al., 2013). At the same time, remote associations are also influenced by intelligence (Lee and Therriault, 2013; Lee et al., 2014). The RAT entails a short and objective scoring time in contrast to the time-consuming and subjective scoring of divergent thinking tasks. In addition, RAT questions are relatively easy to compile, which is conducive to the mass production of tests. This prevents test questions from being exposed in advance, which invalidates them, whereas this often happens with insight problem solving tasks. The RAT provides an objective and convenient way to measure creativity, which has led to its wide use as a tool to evaluate individual creativity (Jen et al., 2004; Huang et al., 2012; Chang et al., 2016).

In recent years, the number of empirical studies of the evaluation of individual creative performance via the RAT has been increasing. Creativity has different levels (Rhodes, 1961; Gabora, 2010; Simonton, 2010; Sowden et al., 2014). Determining which level of creativity the RAT is utilized to measure, the number of versions that have been developed and made available, and how it can be combined with different creativity-related research orientations and technologies are of great importance to creativity-related research. However, very little is known about these. Deeper knowledge and understanding about RAT will help researchers determine how RAT can best be used. Therefore, this study aims to summarize how RAT has been used in a variety of creativity-related studies through an extensive review of the literature, based on which the relationship between the RAT and creativity is established.

## Creativity and Remote Association

Many theories have been advanced regarding the creative process, including the BVSR model (blind-variation and selective-retention process) (Simonton, 2010), dual-process theory (Sowden et al., 2014), and associative theory (Mednick, 1962). These theories all attempt to explain how individuals produce creativity via thinking, explore the mechanism of different stages in the process, and emphasize the benefits of divergent associations for creativity generation, examining, for example, how individuals change the direction of thinking and retain original concepts in the creative process (Campbell, 1960), how defocusing and focusing attention affects creativity generation (Gabora, 2010), and how the transformation of automation and control processes can enhance creativity (Sowden et al., 2014), connect seemingly unrelated elements, and form new relations among them in order to meet specific needs or purposes, i.e., remote association (Mednick, 1962).

Mednick (1962) explains individuals' different creative abilities through the associative hierarchy, holding that highly creative people have a flat associative hierarchy and relatively good remote associative ability, through which they are able to produce unusual and novel ideas. On the contrary, less creative people have a steep associative hierarchy, through which they produce close associations. Meanwhile, independent concepts are associated by individuals through serendipity, similarity, and mediation. Serendipity is the unexpected formulation of novel ideas under continuous stimuli from the environment, such as the discovery of electricity. On the other hand, highly analogous concepts are linked to each other through similarity, such as the rhymes of lyrics. Lastly, mediation is a way of linking concepts via their common target words, such as the word “snake.” Although empirical research has not fully supported the associative theory (Benedek and Neubauer, 2013), it was still found that highly creative people have higher fluency and originality. One study analyzed the conceptual connection of high and low creatives, finding that the semantic networks of high creatives are less rigid and separated into less subcommunities (Kenett et al., 2014). Subsequent research, combined with the perspective of network topologies, pointed out that the semantic networks of highly creative people have higher connectivity, smaller distance, and lower community and are more flexible (Kenett and Faust, 2019). These results showed the flexibility and originality of the conceptual ideas of high creatives in the associative process. It can be seen that the remote association system influences the core ability of individual creativity. In this aspect, remote association can be considered a core ability that affects individual creativity.

## The Development and Application of the Remote Associates Test

To assess an individual's remote associative ability, Mednick (1968) compiled and developed the RAT based on the associative theory. Researchers often select three remotely associated stimulus words that are commonly seen and familiar to participants and ask them to think of a word that can be linked to all three stimuli words. For instance, for the stimuli words “blood,” “music,” and “cheese,” the word “blue” can be paired with all three to create three compound words: “blue blood,” “blue music,” and “blue cheese.” The RAT consists of 30 questions, and participants are given one point for each correct answer, and zero for each wrong one. The participant's total score represents their remote associative ability.

However, the type of creativity that the RAT assesses remains controversial. The RAT is generally regarded as a convergent thinking test (Lee and Therriault, 2013; Lee et al., 2014) and is significantly related to typical insight problem solving (Huang et al., 2012; Chang et al., 2016). Bowden and Jung-Beeman (2003a) described the three ways of association often used to compile RAT questions, which are (1) synonymy, (2) formation of a compound, and (3) semantic association. The combination of a stimulus word and a target word having the same meaning, such as “same” and “match,” is called *synonymous association*. Meanwhile, compound words are formed when the stimulus and the target word can be combined to form a compound, such as “match-head.” Lastly, semantic association occurs when the stimulus and the target word can be associated based on their meaning, such as “tennis match.” Moreover, Bowden et al. noted that the words in a single RAT question could be associated in several ways. Therefore, for research on insight problems, a standardized database of 144 RAT questions was compiled based on how compounds are formed. In addition, both the RAT and the intelligence test evaluate analytical thinking and are positively correlated with the Wechsler Adult Intelligence Scale and the Raven test (Lee and Therriault, 2013; Lee et al., 2014). This indicates that the individual's performance on the RAT is also influenced by intelligence. On the other hand, based on the view of associative thinking (Benedek et al., 2012), a few studies have pointed out that the process of RAT problem-solving involves divergent thinking (Wu et al., 2016) and found that RAT scores were positively correlated with divergent thinking (Wu et al., 2017). In summary, the RAT demonstrates the diversity of the creative process, involving the integration of divergent, convergent, and analytical thinking.

Jen et al. (2004) were the first to compile RAT questions via “word-pairing,” similar to how semantic compounds (Mednick, 1968) are formed, for Chinese native speakers, which they developed into the Chinese RAT. Their study set a precedent whereby Chinese people use RATs to evaluate individuals' creativity. After that, the Chinese Word Remote Associates Test (CWRT) of Huang et al. (2012) and the Chinese Radical Remote Associates Test (CRRAT) of Chang et al. (2016) were developed. The CWRT is compiled based on semantic association, while the CRRAT was based on radical pairing. In summary, three versions of the Chinese RAT were developed based on Chinese radical pairing, Chinese character pairing, and pairing of Chinese character compounds (no fewer than two Chinese characters).

Benefiting from its simple preparation of items and its short and objective scoring time, the RAT not only overcomes the shortcomings of insight problems, which are easily affected by the exposure of the test questions but also provides more stimulus needs for psychological experiments, especially in behavioral experiments (Wu and Chen, 2017) and cognitive neuroscience experiments (Bowden and Jung-Beeman, 2003a). Hence, the RAT has been widely used in various fields, including the exploration of the process of insight problem solving (Huang, 2017), the evaluation of individual creativity (Baer and Kaufman, 2008), as a reference for diagnosing mental illness (Heatherton and Vohs, 2000; Vohs and Heatherton, 2001; Tu et al., 2017), as tools to explore the process whereby creativity develops in cognitive neuroscience (Wu et al., 2016, 2019), and for the analysis of characteristics that might affect the difficulty of RAT questions (Bowden and Jung-Beeman, 2003a; Hung et al., 2016), investigating the relationship between intrapersonal and interpersonal relationships and creative thinking performance (Colzato et al., 2013). All these show that the RAT has been commonly used to explore different orientations of creativity, from process, person, and product to place.

## The Present Study

This study reviews empirical studies of creativity that employed RAT. By analyzing the results of studies published from 2000 to 2019, the present study intends to illustrate how RAT was applied to different research of creativity and the versions of RAT developed in these studies. The analysis and collation will help construct a framework that will show the practical application of RAT to the theory of creativity.

## Methods

### Paper Selection

Papers pertaining to RAT published between 2000 and 2019 were retrieved from the SCOPUS database in November 2019. SCOPUS provides a large number of abstracts and references from peer-reviewed journals and highly influential research papers. By November 2019, SCIE and SSCI journals had been incorporated into SCOPUS. To ensure research quality, the retrieved and selected papers were mainly written in English, for the authors of this study have a limited understanding of other languages.

The retrieval and collation of the research papers consisted of two stages. During Stage One, three keywords were keyed in to search and retrieve papers, namely, “remote association,” “remote associates test,” and “remote associate.” In addition, the Boolean operator “or” was used to obtain the union of the three sets of keywords. In total, 256 papers were retrieved for collation.

In Stage Two, three researchers systematically selected the retrieved papers to be included in the present study according to the title and abstract based on the following criteria: (1) The research tools include the RAT, (2) the research topic is creativity, (3) the paper is a data-based empirical research, and (4) an electronic or printed version of the full text is available. If the abstract did not provide sufficient information for collation, the researchers carefully read the main parts of the paper (i.e., research methods and results). A total of 172 of the 256 retrieved papers met the above criteria and were included in the systematic review.

### Coding Procedure

The content analysis was carried out in several steps. First, the selected papers were coded according to the purpose of the RAT usage to identify their research orientations. At present, the most comprehensive framework of creativity-related research is the 4P's Model of Creativity (Rhodes, 1961), which consists of the novel and appropriate products created by individuals, the personality of a creative person, the environment that enables an individual to produce creativity, and the process whereby an individual develops creativity. Accordingly, the selected papers were divided into nine categories based on their research orientation, which are (1) remote association, (2) insight problem solving, (3) general creative process, (4) test development, (5) individual difference, (6) effect of treatment, (7) clinical case, (8) social interaction effect, and (9) predictor or criterion.

Remote association analyzes the remote associative process and its influencing factors; insight problem solving assesses the process of insight problem solving and its influencing factors; the general creative process involves aspects of the creative process separate from remote association or insight. Test development involves establishing new versions of the RAT or exploring test question compilation. Consequently, individual difference explores the diversities in RAT performance of various groups of participants. Effect of treatment tests the effects of different experimental interventions on RAT performance, and clinical case examines the RAT performance of individuals with various mental disorders. Social interaction effect, on the other hand, determines the influence of cultural experience or interpersonal relationships on RAT performance, while predictor or criterion is pertinent to the relationship between individual RAT performance and certain variables.

These nine categories are extended from the 4P model. First, in the process orientations, the RAT was developed based on the associative theory of creativity (Mednick, 1962), which aimed to analyze how individuals combine unrelated elements into a new relationship. At the same time, the RAT is considered to have a similar cognitive process as insight problem solving and is often used as an insight problem (Bowden and Jung-Beeman, 2003a). In addition, most studies regard the RAT as a general measure of creativity and do not emphasize remote association or insight problem solving. Therefore, the process orientation includes three themes, namely, remote association, insight problem solving, and general creative process. Second, in the product orientation, this research focuses on the development of the RAT and the exploration of its internal components. There is only one theme: test development. Third, in the personality orientation, apart from dividing the two themes of innate individual differences and acquired intervention, we specifically examine the similarities and differences in creativity between clinical cases and typical developers. Finally, in the place orientation, we explore the role of creativity both interpersonally and intrapersonally. Thus, there are two themes: social and cultural effects, and predictor or criterion.

**Figure 1:**
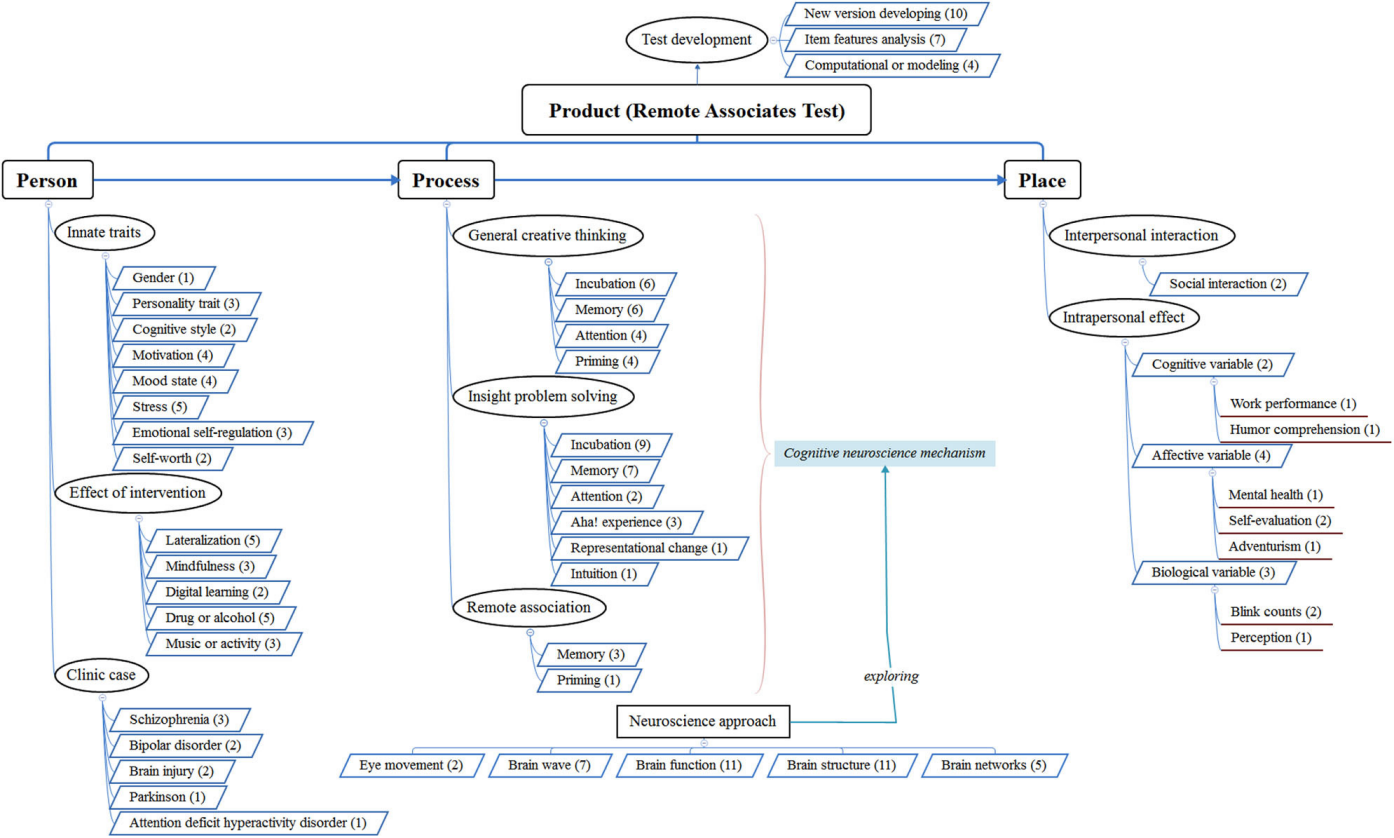
The framework of the remote associates test applied to the 4P creativity model. Only some subcategories of each category are presented.

The researchers recorded the author, keywords, and version of RAT used by each paper, its research orientation (i.e., behavioral research, cognitive neuroscience, or modeling), and the journal in which it was published. The records are shown in the Appendix in Supplementary Material, which includes the research purpose, topic, subtopic, RAT version, methodology, and research orientation of each paper. Lastly, a framework is established based on the nine categories of creativity-related research conducted via RAT (shown in Figure 1). To ensure the reliability of coding, the three researchers reconciled coding differences through discussion, and each paper was labeled with one main research topic and subtopic.

## Results and Discussion

### Overall Findings

In this study, 172 of the 256 papers retrieved from the literature database SCOPUS were chosen to systematically review how RAT was used to investigate creativity from 2000 to 2019. This section presents a comprehensive introduction to the 172 papers in terms of the publication date, research topic, research method, and RAT version and the journals in which these papers were published. The implications of each research topic and the major findings are discussed in the next part.

**Figure 2:**
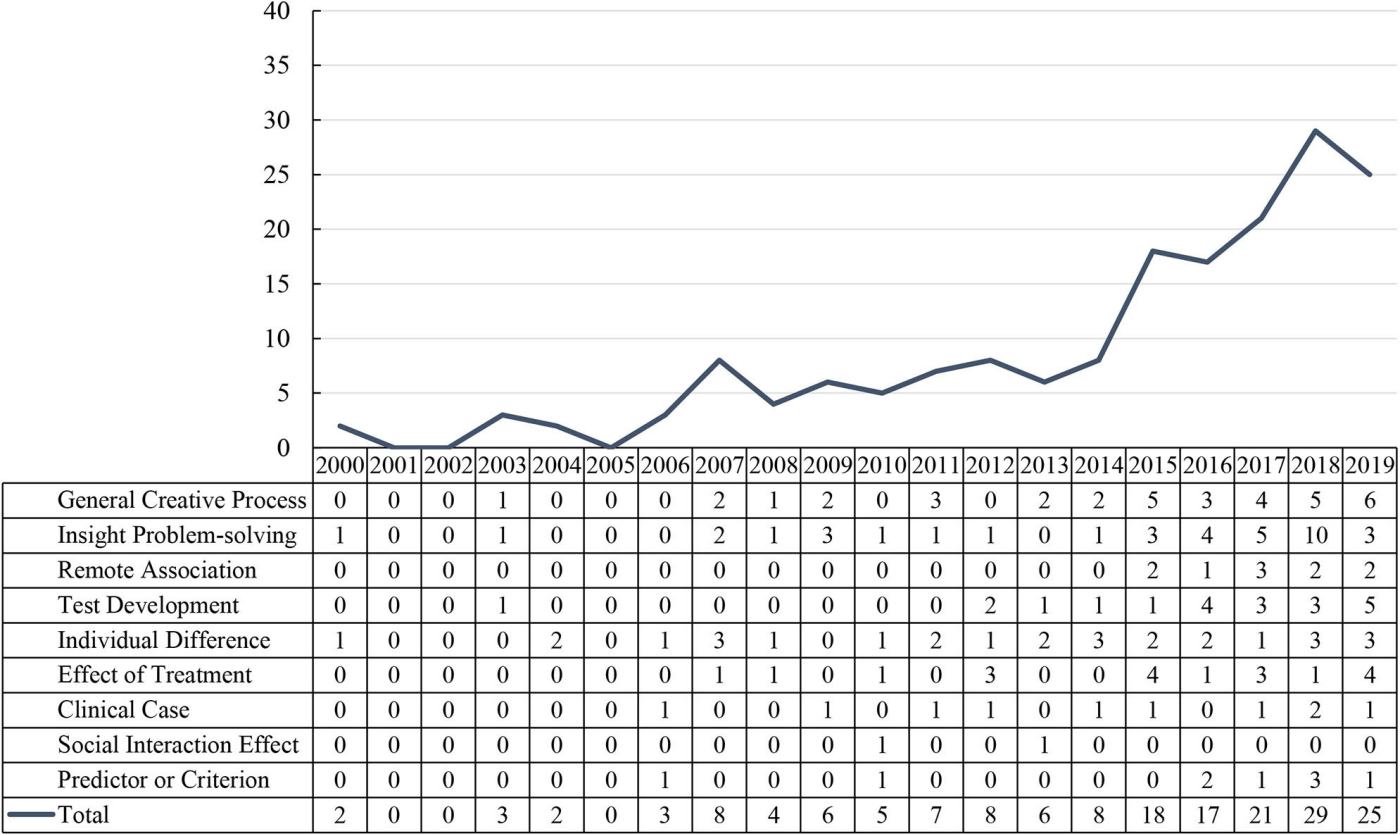
Number of studies in each creativity-related research category from 2000 to 2019.

As shown in Table 1 and Figure 2, there were more than 20 papers published in 2017, 2018, and 2019 (21, 29, and 25, respectively), while in 2015 and 2016 there were more than 10 per year (18 and 17, respectively). Moreover, the years 2007, 2012, and 2014 each saw eight papers, seven in 2011, six each in 2009 and 2013, five in 2010, three each in 2003 and 2006, two each in 2000 and 2004, and none in 2001, 2002, and 2005. These numbers show that RAT has been used by a growing number of researchers in the past five years, suggesting that researchers have come to recognize its value.

**Table 1:** Number of reviewed papers by year of publication.

**Year**	***n***	**Year**	***n***
2000	2	2010	5
2001	0	2011	7
2002	0	2012	8
2003	3	2013	6
2004	2	2014	8
2005	0	2015	18
2006	3	2016	17
2007	8	2017	21
2008	4	2018	29
2009	6	2019	25

The research orientation of each paper was then analyzed. Out of 172 selected papers, 128 reported behavioral research, 41 were related to cognitive neuroscience, and 3 explored the association between RAT and creativity with computational models (modeling). Regarding the number of papers per academic journal, *Creativity-Related Research Journal* ranked first, in which 13 of the 172 papers were published (*n* = 13); *Frontiers in Psychology* ranked second (*n* = 11), followed by *Journal of Creative Behavior* (*n* = 10), *Memory and Cognition* (*n* = 6), *Neurosymbologia* (*n* = 6), *Thinking Skills and Creativity* (*n* = 5), *Cognition* (*n* = 5), and *Personality and Individual Differences* (*n* = 5). The academic journals publishing less than five of the papers are listed in the Appendix in Supplementary Material.

**Figure 3:**
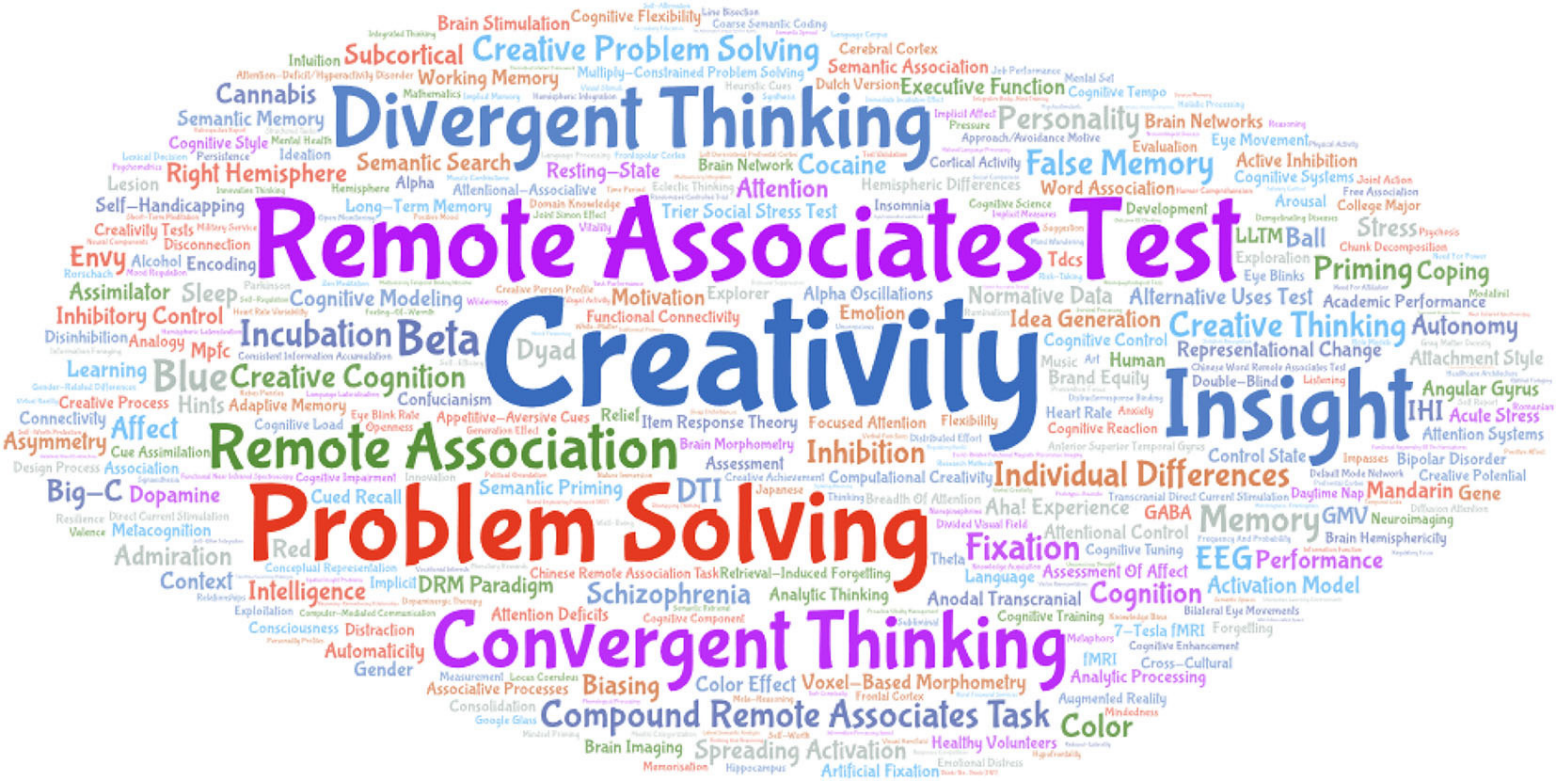
The number of keywords in the creativity-related research using the remote associates test from 2000 to 2019.

**Figure 4:**
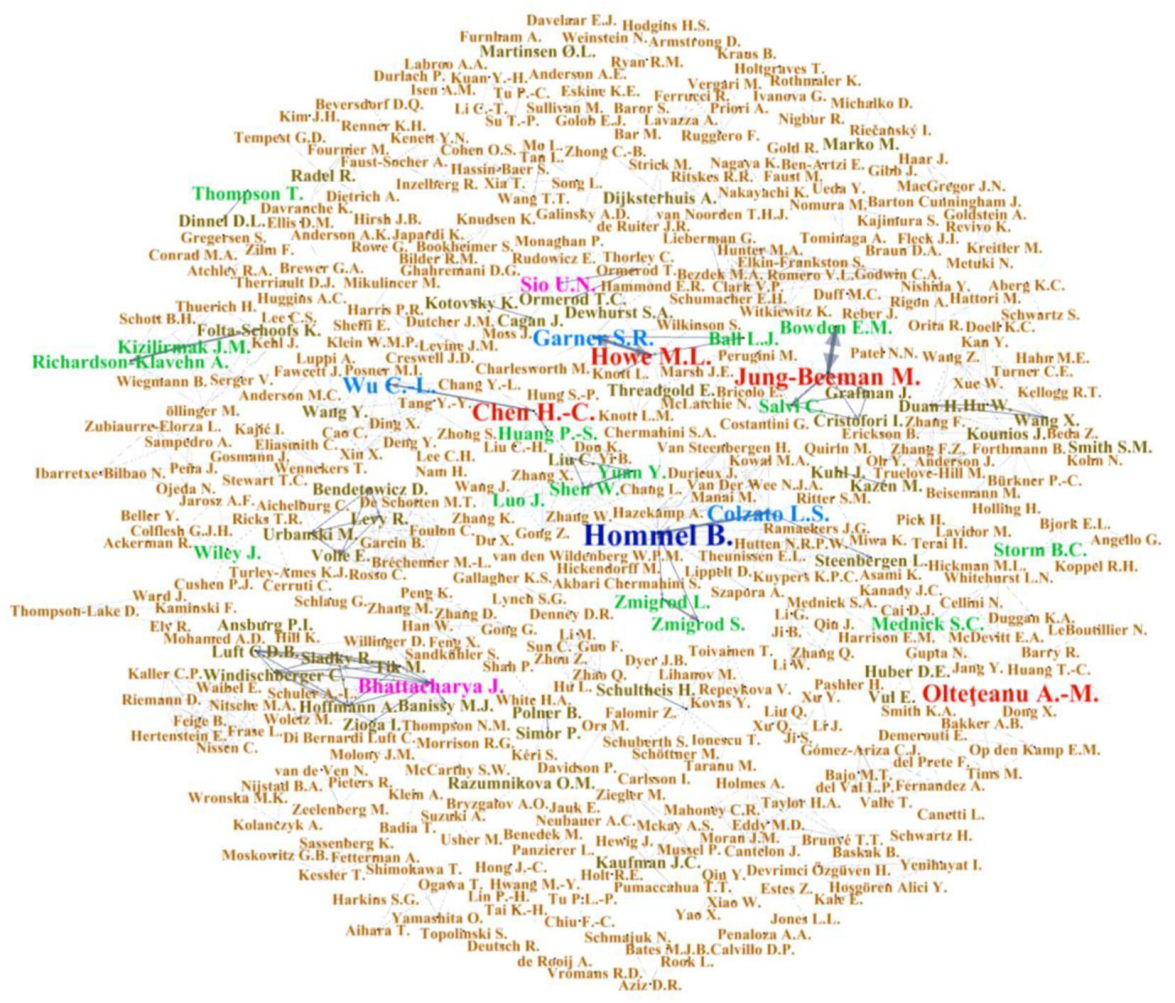
Collinear network map of authors of creativity-related research using the remote association test from 2000 to 2019.

Furthermore, the keywords of these 172 articles were analyzed. Results showed that Creativity (76) is the most frequent, followed by Remote Associates Test (41), and the keywords with more than 10 occurrences are Problem Solving (38), Insight (31), Divergent Thinking (23), Convergent Thinking (21), and Remote Association (13). This shows that most studies use RAT to explore topics such as creativity, problem solving, or insights. The results are shown in Figure 3. In addition, we analyzed the connections between the author groups of these articles and displayed the connections between authors with a collinear network map (see Figure 4). The results indicate that Hommel B. of Leiden University has published the most articles (nine) in the past 20 years, while Howe ML of the University of London, Olteteanu A.-M. of Freie Universität Berlin, Jung-Beeman M. of Northwestern University, and Chen H.-C of the National Taiwan Normal University have published six articles each, and Bhattacharya J. of Goldsmiths University of London has published four articles. All these authors urge their research teams to collaborate and publish, forming a closely connected network. In addition, most of the research conducted by researchers using RAT forms a single network without too many cross-domain connections.

In terms of RAT versions, most studies adopted the Compound Remote Associates Problems (Bowden and Jung-Beeman, 2003a) (*n* = 56) and RAT (Mednick, 1968) as the research tool (see Table 2). The difference between the two is that the Compound Remote Associates Problems emphasizes the connection of a stimulus and a target word (the answer to a RAT question) to form compound words, while the RAT includes but is not limited to the formation of a compound. In addition, 25 studies used the Chinese versions of RAT, 9 used German versions, 8 Dutch versions, and a total of 7 studies applied the computerized RAT. In addition, six studies used the RAT in Hebrew, five in Japanese, two each in Hungarian, Italian, and Spanish, and one each in French, Korean, Norwegian, Polish, Romanian, Russian, Slovak, and Turkish.

**Table 2:** The number of RAT versions.

**Version**	***n***
Compound Remote Associates Problems	56
RAT	46
Chinese version of the RAT	25
German version of the RAT	9
Dutch version of the RAT	8
Computerized version of the RAT	7
Hebrew version of the RAT	6
Japanese version of the RAT	5
Hungarian version of the RAT	2
Italian version of the RAT	2
Spanish version of the RAT	2
French version of the RAT	1
Korean version of the RAT	1
Norwegian version of the RAT	1
Polish version of the RAT	1
Romanian version of the RAT	1
Russian version of the RAT	1
Slovak version of the RAT	1
Turkish version of the RAT	1

### The Nine Categories of Creativity Studies Applying the RAT

As stated above, the 172 studies on creativity that employed RAT were divided into nine categories (remote association, insight problem solving, general creative process, test development, individual difference, effect of treatment, clinical case, social interaction effect, and predictor or criterion). Table 3 and Figure 1 illustrates how RAT was used in the studies on creativity to explore these nine categories. In this section, the details for each topic are explained.

**Table 3:** The number of the studies for each dimension.

**Dimension**	***N***
Insight problem-solving	37
General creative process	36
Individual difference	28
Test development	21
Effect of treatment	19
Clinical case	10
Remote association	10
Predictor or criterion	9
Social interaction effect	2

#### Remote Association

The RAT (Mednick, 1968), which originated from the associative theory of creativity, aims to measure individuals' remote associative ability. Later, Bowden and Jung-Beeman (2003a) adapted it as the Compound Remote Associates Problems. It is used to evaluate the cognitive process of individuals in solving insight problems. However, most researchers using RAT only regarded it as a tool to measure creative thinking and overlooked its ability to evaluate remote association or insight problem solving. Therefore, this study divided the papers on creative thinking process into three categories: (1) remote association, (2) insight problem solving, and (3) general creative process.

Out of the 172 researches, ten studies explored the remote associative process. These studies mainly focused on how memory affects remote association, such as semantic search, memory retrieval, and prior knowledge (Davelaar, 2015; Klein and Badia, 2015; Kajić et al., 2017), and on aspects of the brain mechanisms underlying the formation of remote associations, such as brain networks, brain structure, brain function, and brain waves (Wu et al., 2016; Bendetowicz et al., 2017, 2018; Di et al., 2018; Pick and Lavidor, 2019; Zhou et al., 2019). In addition, some studies examined the effects of priming on remote association (Sassenberg et al., 2017). Compared with the general creative thinking process or insight problem solving process, the RAT has been rarely used to explore individuals' remote associative ability in recent years. Most regarded individuals' RAT performance as their creative thinking or insight problem solving ability, rather than their remote associative ability. Regardless, some studies explored the effects of memories and priming on remote association and the mechanisms that occur in the brain when one is making remote association. This suggests that remote association still receives much attention. On the other hand, empirical research exploring remote association or associative theory does not use the RAT (e.g., Benedek and Neubauer, 2013). The reason is that most studies only collect scores on the RAT and seldom collect individuals' responses during the RAT problem solving.

#### Insight Problem Solving

A total of 37 studies explored the process of insight problem solving using RAT, including how individuals' memory (e.g., false memory) affects their developing insight (Howe et al., 2010, 2011, 2016; Garner and Howe, 2014; Kizilirmak et al., 2016b; Ellis and Brewer, 2018; Howe and Garner, 2018), the incubation mechanism, such as dreams (Sio and Rudowicz, 2007; Vul and Pashler, 2007; Cai et al., 2009; Kohn and Smith, 2009; Penaloza and Calvillo, 2012; Nam and Lee, 2015; Sio and Ormerod, 2015; Morrison et al., 2017; Sio et al., 2017), how representational change affects one's insight problem solving (Barton et al., 2009), the aha! experience of insight (Bowden and Jung-Beeman, 2003b; Du et al., 2017; Kraus and Holtgraves, 2018), the mechanisms that occur in the brain when solving insight problems, such as brain networks, brain structure, brain function, and brain waves (Sandkühler and Bhattacharya, 2008; Kizilirmak et al., 2016a; Shen et al., 2016b; Rothmaler et al., 2017; Erickson et al., 2018; Ji et al., 2018; Ogawa et al., 2018; Ruggiero et al., 2018; Tik et al., 2018; Tempest and Radel, 2019), and eye movements (Huang, 2017; Huang et al., 2019). In addition, some studies focused on how individuals' attention (Cushen and Wiley, 2018; Zmigrod et al., 2019), meta-cognition (Storm and Hickman, 2015), creative thinking fluency (Ansburg, 2000), and intuition (Kizilirmak et al., 2018) influence insight problem solving. The RAT is often used to measure individuals' insight problem solving ability as well as to test the internal cognitive process (like incubation and the aha! experience) and physiological mechanisms (like brain function and brain waves) during insight problem solving. After the compound remote associates problems were developed, they were widely used to explore insight problem solving topics, especially in cognitive neuroscience.

#### General Creative Process

A total of 36 papers on the general creative thinking process employed RAT. Studies on the general creative thinking process explored how the incubation mechanism (such as sleep, fixation, inhibition, and dreaming) affects creative thinking (Zhong et al., 2008; Sio et al., 2013; Smith et al., 2013; Koppel and Storm, 2014; Whitehurst et al., 2016; Carlsson et al., 2019), how individuals' memory, such as working memory and memory retrieval, affects creative thinking (Ricks et al., 2007; Dewhurst et al., 2011; Storm et al., 2011; Gómez-Ariza et al., 2017; Beda and Smith, 2018; Wang et al., 2019), how attention span enhances the output of creative ideas (Ansburg and Hill, 2003; Schmajuk et al., 2009; Zmigrod et al., 2015; Wronska et al., 2018), the association between creative thinking and how the brain works (such as brain networks, brain structure, brain function, and brain waves) (Razumnikova, 2007; Cerruti and Schlaug, 2009; Brunyé et al., 2015; Aberg et al., 2017; Godwin et al., 2017; Colzato et al., 2018; Dong, 2018; Hertenstein et al., 2019; Li et al., 2019; Peña et al., 2019; Schuler et al., 2019), and how priming (Moss et al., 2011; Chiu and Tu, 2014; Radel et al., 2015; Baror and Bar, 2016), metacognition (Ackerman and Beller, 2017), analogy (Jones and Estes, 2015), colors (Xia et al., 2016), genes (Han et al., 2018), and intelligence (Mussel et al., 2015) influence creative thinking. In brief, the RAT has been widely used to evaluate individuals' creative thinking ability and to explore the influence of various factors on the creative thinking process and creative performance.

In integrating the items of the process orientation, previous studies comprehensively explored the influence of factors such as memory, gestation, attention, and triggering on the general creative process. In contrast, only a few studies have explored remote association in terms of memory and motivation. It is worth mentioning that the unique core factors of typical insight problem solving (i.e., representation transformation, the “aha” experience) have only been explored in research on this topic, showing the difference between insight problem solving and the other two categories of process orientation. Cognitive neuroscience approaches (e.g., brain network, brain structure, brain function, brain waves) are commonly used to explore three creative processes, showing that the examination of creative processes at the neural level is universal in creativity research.

#### Test Development

Twenty-one studies focused on RAT development, of which 10 developed different versions of RAT or the RAT in different languages (Bowden and Jung-Beeman, 2003a; Akbari et al., 2012; Terai et al., 2013; Salvi et al., 2016a,b; Xiao et al., 2016; Wu and Chen, 2017; Orita et al., 2018; Olteteanu et al., 2019b; Toivainen et al., 2019). Eight studies explored the test questions with the goal of providing a reference for question compilation (Lee et al., 2014; Hung et al., 2016; Olteteanu and Schultheis, 2017; Wu et al., 2017; Marko et al., 2018; Beisemann et al., 2019; Wu, 2019). Moreover, four studies used computational methods to simulate individuals' performance on RAT and investigated possible influencing factors on creativity (Gupta et al., 2012; Olteteanu and Falomir, 2015; Olteteanu et al., 2018, 2019a). In summary, the researchers analyzed the characteristics of RAT questions to further improve them and developed RAT versions in different languages that can be applied to non-English speakers. In this way, it is hoped that the usability and popularity of the RAT will increase.

Recently, researchers have developed a visual version of the RAT (Toivainen et al., 2019) that enhances participants' imagination using image stimulus to break through past limitations of using language as a stimulus and to break down the barriers between different languages so as to make cross-cultural comparisons. However, further research is needed as to whether an individual's performance in this version is not influenced by verbal intelligence. In addition, based on the findings for the process orientation, computational science research allows us to directly examine the entire problem-solving process with simulation technology (Olteteanu and Falomir, 2015; Olteteanu et al., 2018, 2019a). This research approach can also enhance the understanding of the influence of various item components on performance, provide a reference for test item preparation, and even aid in the development of an adaptive RAT. In addition, through the exploration of the entire problem-solving process, follow-up research may be able to identify the differences between creative thinking problem-solving and typical problem-solving in more detail.

#### Individual Difference

A total of 28 studies investigated individual differences using RAT as a tool. The similarities and differences in creative thinking among individuals with different cognitive functions or mood states are explored, of which emotional factors account for the majority. Nineteen studies explored the effect of affective variables on creative thinking, of which five examined the effect of stress (Renner and Beversdorf, 2010; Creswell et al., 2013; Marko, 2016; Duan et al., 2019a,b), four focused on individuals' motivations (van de Ven et al., 2011; Rook, 2014; Martinsen and Furnham, 2015; de Rooij and Vromans, 2018), four on the importance of mood states (Mikulincer and Sheffi, 2000; Isen et al., 2004; Rowe et al., 2007; Schwartz and Canetti, 2014), three investigated the influence of emotional self-regulation (Topolinski and Deutsch, 2012; Knott et al., 2014; Kazén et al., 2015), two examined the effect of individuals' self-worth (Thompson and Dinnel, 2007a,b), and one evaluated the effect of extrinsic rewards (Cristofori et al., 2018). These studies reveal the role that affective factors (stress, motivation, and emotion) play in creative thinking.

On the other hand, some studies explored creativity and gender differences using RAT (Razumnikova and Bryzgalov, 2006), while others considered individual cognitive styles, such as administrative and legislative styles (Ward et al., 2008; Salvi et al., 2016b), personality traits (Thompson, 2004; Martinsen, 2011; Kaufman et al., 2013), proficiency (Zilm et al., 2019), creative ability (Japardi et al., 2018), and sleep hours (Simor and Polner, 2017). In these studies, participants were grouped based on their test scores to compare and contrast their differences. These surveys indicate that researchers emphasized how individual background knowledge affects creativity.

#### Effect of Treatment

Nineteen studies explored how experimental interventions affect creative performance. Five studies manipulated the left and right visual fields to understand how lateralization affects creative thinking (Bowden and Jung-Beeman, 2007; Kuhl and Kazén, 2008; Goldstein et al., 2010; Gold et al., 2012; Turner et al., 2017). Five studies investigated the influence of certain experimental interventions on creative thinking, such as e-learning (Hong et al., 2019; Huang, 2019) and mindfulness (Strick et al., 2012; Kim, 2015; Colzato et al., 2017). In addition, some studies explored how drug (Ding et al., 2015; Mohamed, 2016; Hutten et al., 2019) and alcohol use (Jarosz et al., 2012; Benedek et al., 2017) influence creative thinking, and whether music (Eskine et al., 2018; Threadgold et al., 2019), eye movements (Fleck and Braun, 2015), and outdoor sports (Ferraro, 2015) promote creative performance. Generally, the aforementioned studies focused on the effects of external manipulation and interventions on creative thinking.

#### Clinical Case

Ten studies examined the differences in creative thinking between clinical cases of schizophrenia (Suzuki and Usher, 2009; Armstrong, 2012; Polner et al., 2018), bipolar disorder (Tu et al., 2017; Hoşgören et al., 2019), brain injury (Kowal et al., 2015), Parkinson's disease (Faust-Socher et al., 2014), attention deficit hyperactivity disorder (ADHD) (White and Shah, 2006), and other disorders (Denney et al., 2011; Rigon et al., 2018) and individuals with typical development, showing that the performance of clinical cases in the RAT has also received attention. However, not every mental illness has shown differences in creative thinking, resulting in relatively few studies on this topic.

Integrating the literature on the personality orientation reveals that at the level of innate internal factors, the relationship between emotion and creative cognition has been highly emphasized in the past 20 years, while the influences of other cognitive factors have received less attention. However, benefiting from emerging technologies, cognitive neuroscience technology can provide further evidence on how individuals with different cognitive functions (e.g., executive functions, academic majors) influence creative thinking and other issues. In addition, longitudinal studies or cross-sectional studies comparing different age groups are relatively rare, making it difficult to understand the development of remote association ability. At the acquired intervention level, the results showed that the RAT is generally used as an indicator for intervention. In long-term teaching training or short-term experimental manipulation, cognitive function training, or physiological manipulation, internal or external intervention is helpful to the performance of RAT. It is worth mentioning that with the convenience of information technology, digital learning can not only provide more continuous creative thinking training but also record individual learning process, allowing us to better understand the process of change of individual creative thinking and accurately evaluate the corresponding effectiveness of the teaching program. Finally, the number of clinical case studies is relatively small, probably because not all experts in the clinical field pay attention to creative thinking. This also reflects the importance of cross-domain cooperation. Through the cooperation of clinical, special education, creativity, cognitive neuroscience, and other fields of expertise, it may be possible to further explore the differences between clinical cases and typical developmental individuals in creative thinking at the neurophysiological level.

#### Social Interaction Effect

Only 2 of the 172 studies explored the impact of interpersonal interaction, examining the correlation between one's interaction quality and his/her creative performance (Weinstein et al., 2010; Colzato et al., 2013). Clearly, the RAT has rarely been used to explore the influence of social culture or interpersonal interaction on creative thinking.

#### Predictor or Criterion

Nine studies analyzed the association between individuals' RAT performance and certain abilities and traits, including blink counts (Chermahini and Hommel, 2010; Ueda et al., 2016), humor (Wu and Chen, 2019), work performance (Op den Kamp et al., 2018), perception (Zmigrod and Zmigrod, 2016), mental health (LeBoutillier and Barry, 2018), adventurism (Shen et al., 2018), and self-evaluation (Harkins, 2006; Nagaya and Nakayachi, 2017). These studies revealed the correlation between creativity and other variables, which indirectly shows the impact of creativity on individuals' performance.

The number of the studies using RAT to explore the relationship between creativity and intrapersonal traits or interpersonal interaction is less than that of other categories. The reason may be that the RAT score is regarded as a problem-solving ability. When investigating the relationship between creativity and the place orientation, researchers may prefer to choose creative personality, creative tendency, or other creativity tests for cognitive orientations (e.g., divergent thinking tasks). At the same time, the initial development of RAT was intended for exploring the creative process, and its measurement attribute is different from other creativity tasks. However, this does not mean that the RAT is not suitable for exploring the relationship between creativity and the place orientation. Interpersonal interaction can also be regarded as problem solving, so it is meaningful to explore the relationship between the two.

### The Theoretical Framework of Creativity Studies Applying Remote Associates Test

Integrating the content of the nine categories and the 4P model (Rhodes, 1961), we construct a theoretical framework for creativity research using the RAT to understand how the RAT has been used in the past 20 years. It is applied as inquiries on the theme of creativity and may serve as a reference for future directions of research, as shown in Figure 1. This framework, taking RAT as its core, demonstrates that empirical studies using the RAT explore the influences of individual differences, internal processes, and interactions with the environment on creative thinking.

First, from the Product perspective, RATs were developed following internal and external approaches. The former involves an analysis of the item components, and the latter extends the approach to versions in different languages. Moreover, in cooperation with computational science experts, computer science technology was used to model the generation of RAT. Second, from the Person perspective, the individual differences in creative thinking are explored along the dimensions of innate traits, interventions, and clinical cases. Furthermore, from the Process perspective, it includes three aspects: general creative thinking, insight problem solving, and remote association. The effect of memory has received attention across these aspects, as have the influences of priming, attention, and incubation on the internal process. In addition, intuition, representational change, and aha! experiences may be the specific issues involved in insight problem solving. The cognitive neuroscience approaches explore the internal process of creative thinking based on a variety of physiological evidence, such as eye movement, brain waves, brain function, brain structure, and brain network. Finally, from the Place perspective, it concerns how remote association capacity affects the interaction between the individual and the environment, including interpersonal interaction and intrapersonal traits. The latter are further divided into cognitive, affective, and biological level variables.

From this model, creativity research using the RAT has focused on the process orientation and applied cognitive neuroscience technology during the past 20 years. However, there are still relatively few empirical studies of topics such as clinical cases, interpersonal interactions, and even how remote association capacity or creative thinking affects individual intrinsic traits. There remains a need for cooperation with experts in clinical and social psychology and other fields to expand the application of RATs.

### Research Trend of Remote Associates Test Applied to Creativity-Related Studies

**Figure 5:**
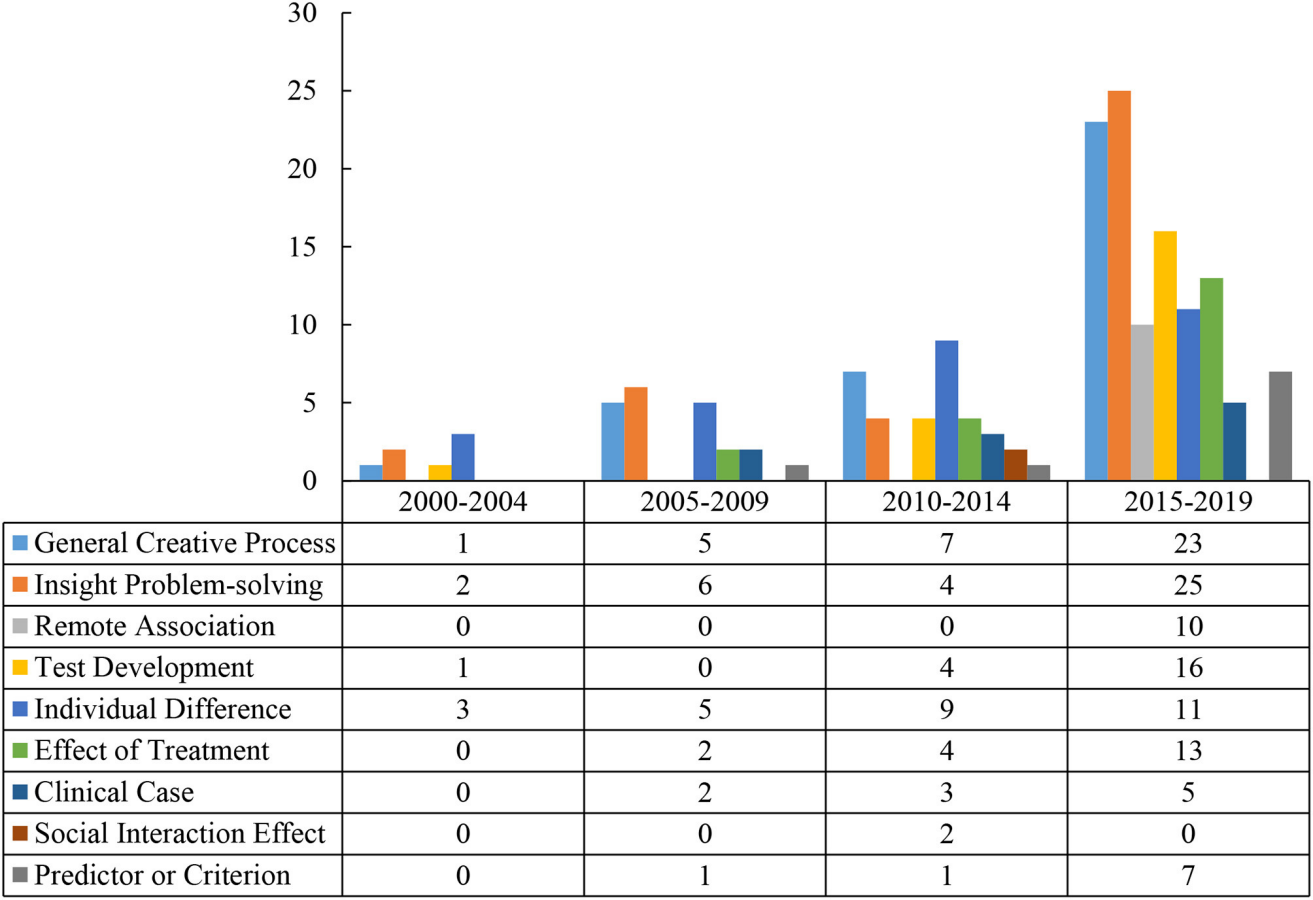
Number of studies in each creativity-related research category every 5 years from 2000 to 2019.

It can be seen from Figure 5 that the number of creativity-related studies that used RAT as a tool increased significantly in 2015. Of these 172 studies, the theme of research during the past 20 years focused on the creative thinking process, including the general creative thinking process, remote association, and the insight problem solving process. In the past 5 years, a significant increase in the research of general creative thinking, insight problem solving, and RAT development can be observed. In addition, studies focusing on the remote association process and the effect of experimental treatments increased in the past 5 years. Moreover, studies that employed RAT to explore creativity in terms of criterion or predictor, and individual difference, have received much attention. On the other hand, no creativity-related research regarding the social interaction effect was published in recent years.

The present study also found that a growing number of creativity-related studies have adopted cognitive neuroscience beginning in 2015, accounting for 85% of the creativity-related studies in the past 20 years. This result accords with the finding that research on the creative thinking process has increased significantly and indicates that cognitive neuroscience technology has been incorporated into creativity-related research. Starting in 2015, some scholars began to explore RAT questions with computational methods, suggesting that RAT development has reached the point that it is amenable to computerized and adaptive-level research. It can be observed that creativity-related research has advanced beyond typical behavioral research.

Lastly, the adaptation of RAT began in 2003 (Bowden and Jung-Beeman, 2003a). It has been successfully translated into Dutch (Akbari et al., 2012), Japanese (Terai et al., 2013; Orita et al., 2018), Italian (Salvi et al., 2016a), Chinese (Shen et al., 2016a; Xiao et al., 2016; Wu and Chen, 2017), and Romanian (Olteteanu et al., 2019b), which suggests that RAT is gaining popularity in countries and regions with a variety of languages.

## Conclusions

The framework of empirical studies using the RAT based on the 4P model demonstrates how this measure can be used to explore the effects of individual differences, internal processes, and personal–environment interactions on creative thinking. The aforementioned results show that most creativity-related research using RAT has focused on insight problem solving, followed by the general creative thinking process, individual differences, test development, and the effects of treatment. Meanwhile, along with the rise of cognitive neuroscience technology in recent years, research on the creative thinking process (general creative thinking process, insight problem solving, and remote association) and test development has increased significantly. However, few creativity-related studies focused on the social interaction effect. In summary, RAT was mostly used to explore the creative thinking process, as it was used when first compiled. Moreover, the present study found that the RAT had been developed in 15 languages, including English, Chinese, German, Dutch, Hebrew, Japanese, Hungarian, Italian, Spanish, French, and Turkish. This reveals the popularity of the RAT across the world. Furthermore, these results suggest that related research on remote association provided a direction for incorporating the cognitive process into creative performance in future creativity-related researches.

### Future Studies

As shown in the collated results, RAT was used to explore various aspects of creativity-related research, which indicates that researchers interpret creativity at different levels. However, the number of studies is not balanced across these domains of the framework, for most of them have focused on the process of creative thinking, which suggests that more attention should be directed to the impact of individual differences (Person) or social interactions (Place) on creative thinking.

The existence of versions of RAT in at least 15 different languages (Akbari et al., 2012; Terai et al., 2013; Shen et al., 2016a; Xiao et al., 2016; Wu and Chen, 2017; Orita et al., 2018; Olteteanu et al., 2019b) suggests its value in creativity-related research. However, the characteristics of each language version of RAT are different. Researchers modified the way the stimuli are associated in order to conform to the language habits of a certain region (Jen et al., 2004; Huang et al., 2012; Chang et al., 2016). The challenge for future studies is to determine which ability the RAT is evaluating for each language version. On the other hand, it is worth mentioning that these versions of RAT in different languages are conducive to cross-cultural comparisons and explorations of the common process of remote association.

In addition, with the popularity of computational science and item response theory, researchers have used high-level statistical techniques to analyze the RAT test items and simulate the performance of participants on different RAT questions. These studies will help researchers master the key points of the test compilation to develop higher-quality test questions, thus improving the intrinsic validity of the test. Moreover, cooperation with computer scientists is conducive to the development of computerized and adaptive RATs. In this way, researchers can more effectively and accurately evaluate individuals' remote associative ability.

The literature review of this study reveals greatly increasing research on the creative thinking process in recent years, including general creative thinking, insight problem solving, and remote association. These research outcomes have been achieved partly because they reach beyond the limitations of behavioral research to explore the creative thinking process from the perspective of objective and micro-physiological mechanisms. These studies measured brain waves, which provided temporal information; adopted functional magnetic resonance imaging to provide spatial information on a specific brain area; and utilized structural magnetic resonance imaging and diffusion tensor imaging to comprehensively understand the association between the brain network and creative thinking. However, only a few creativity-related studies have applied two or more cognitive neuroscience technologies at the same time (Jung-Beeman et al., 2004). The integration of brain information at the temporal and spatial levels will help researchers understand more accurately the neural mechanism in the brain underlying creative thinking; this will facilitate a better understanding of the physiological mechanism of creative thinking process.

For cognitive psychologists, the application of cognitive neuroscience technology and computational science has a relatively high threshold, which necessitates cross-domain collaboration with cognitive neuroscientists, computational scientists, and statisticians. Moreover, cooperation with educators and teachers will facilitate the transformation of research results into teaching materials. In this way, students, parents, and the public will be updated with the latest knowledge on creativity, thus developing new creative thinking skills training courses. Moreover, researchers could work with an educational neuroscience team to evaluate the effect of creativity cultivation from the perspective of neuroplasticity. To conclude, cross-domain cooperation will contribute to creativity-related research.

Finally, this review appeals for future research on the application of creativity. A framework is proposed to understand how creativity-related research has applied different versions of RAT. The framework is intended to show what forms of future research, especially cross-domain cooperation, can be carried out sustainably. However, as mentioned, creativity is a complex and multifaceted concept. In addition to the 4P's Model of Creativity, Sternberg and Lubart (1999) provided seven approaches to creativity that future studies could use (1) mystical, (2) psychoanalytic, (3) pragmatic, (4) psychometric, (5) cognitive, (6) social-personality, and (7) confluence. Future studies could adopt other models, so different findings may be obtained. Furthermore, the following question warrants further exploration: Which ability does the RAT evaluate? Subsequent research can examine whether the RAT evaluates insight problem solving ability, associative ability, or both of them, which are revealed at different stages of creative thinking? It is hoped that relevant theoretically informed reviews will emerge in the near future.

## Data Availability Statement

All datasets generated for this study are included in the article/Supplementary Material.

## Author Contributions

C-LW data collection, data analysis, data interpretation, and writing. S-YH and P-ZC data analysis. H-CC supervise the project. All authors contributed to the article and approved the submitted version.

## Conflict of Interest

The authors declare that the research was conducted in the absence of any commercial or financial relationships that could be construed as a potential conflict of interest.
