# Supplementary material for: A Systematic Review of Creativity-Related Studies Applying the Remote Associates Test From 2000 to 2019
Source: Front Psychol. 2020 Oct 23;11:573432. doi: 10.3389/fpsyg.2020.573432 (PMC7644781; doi:10.3389/fpsyg.2020.573432)
Supplement: Supplementary file 1 [file Table_1.DOCX]

**Appendix.** Analysis of connection to Remote Associates Test in each reviewed study

| **ID** | **Author** | **Published Journal** | **Dimension** | **Sub-dimension** | **Research purposes** | **RAT version** | **Research Method** |
| --- | --- | --- | --- | --- | --- | --- | --- |
| 1 | Aberg et al. (2017) | Cerebral Cortex | General Creative Process | brain function | This study aimed to clarify the potential links between individual creativity, hemispheric asymmetries in associative priming, and hemispheric asymmetries in DA function. | Remote Associates Test | Neuroscience Approach |
| 2 | Ackerman and Beller (2017) | Thinking and Reasoning | General Creative Process | metacognition | This study aimed to expose shared and distinctive cue utilization in meta-reasoning and meta-memory initial and final judgements. | Compound Remote Associate problems | Behavior Research |
| 3 | Akbari et al. (2012) | Thinking Skills and Creativity | Test Development | new version development | This study aimed to develop a Dutch version of the RAT that can measure creative convergent thinking in the Dutch language. | Remote Associates Test | Behavior Research |
| 4 | Ansburg (2000) | Current Psychology | Insight Problem-solving | fluency of thought | This study aimed to identify general problem-solving skills that underlie the production of insight. | Remote Associates Test | Behavior Research |
| 5 | Ansburg and Hill (2003) | Personality and Individual Differences | General Creative Process | attentional effect | This study aimed to assess whether creative and analytic thinkers differ in their ability to record and access cues to solutions that are presented as a distraction. | Remote Associates Test | Behavior Research |
| 6 | Armstrong (2012) | Creativity Research Journal | Clinical Case | schizophrenia | This study aimed to test the hypothesis that the relationships between (a) creative cognition abilities and (b) creative cognition and schizotypy variables would significantly increase the ability to predict creative performance and provide a more accurate survey of the schizotypic creative cognitive advantage. | Remote Associates Test | Behavior Research |
| 7 | Baror and Bar (2016) | Psychological Science | General Creative Process | priming effect | This study aimed to examine whether the activation of associations is state-dependent. | Hebrew version of the RAT | Behavior Research |
| 8 | Barton et al. (2009) | Journal of Creative Behavior | Insight Problem-solving | representational change | This article aimed to propose and test a preliminary classification of insight problems based on several restructuring characteristics, and to compare performance on classic spatial insight problems with two types of verbal tests. | Compound Remote Associate problems | Behavior Research |
| 9 | Beda and Smith (2018) | Memory and Cognition | General Creative Process | memory effect | This study aimed to test the red herring retrieval hypothesis, which states that fixation in creative problem solving is worse when memory is used to store red herrings. | Compound Remote Associate problems | Behavior Research |
| 10 | Beisemann et al. (2019) | Journal of Creative Behavior | Test Development | item features | This study aimed to use LSA as an alternative scoring for the RAT. | German version of the RAT | Behavior Research |
| 11 | Bendetowicz et al. (2017) | Cortex | Remote Association | brain structure | This study aimed to test the hypothesis that brain networks involved during creative performance may also support and relate to individual creative abilities. | Remote Associates Test | Neuroscience Approach |
| 12 | Bendetowicz et al. (2018) | Brain | Remote Association | brain connectome | This study aimed to examine the ability to generate and combine remote semantic associations, in relation to creative abilities, in patients with focal frontal lesions. | Remote Associates Test | Neuroscience Approach |
| 13 | Benedek et al. (2017) | Consciousness and Cognition | Effect of Treatment | alcohol | This study aimed to examine the effects of mild alcohol intoxication on creative cognition in a placebo-controlled design. | Remote Associates Test | Behavior Research |
| 14 | Bowden and Jung-Beeman (2003) | Psychonomic Bulletin and Review | Insight Problem-solving | aha experience | This study aimed to test for an association between semantic activation in the right hemisphere (RH) and left hemisphere (LH) and the aha! experience when people recognize solutions to insight-like problems. | Compound Remote Associate problems | Behavior Research |
| 15 | Bowden and Jung-Beeman (2003) | Behavior Research Methods, Instruments, and Computers | Test Development | new version development | This paper aimed to provide a brief overview of the compound remote associate problems that have been developed and tested and normative data regarding the percentage of participants solving, and the mean time-to-solution for, each problem at each time limit. | Compound Remote Associate problems | Behavior Research |
| 16 | Bowden and Jung-Beeman (2007) | Methods | Effect of Treatment | lateralization | This study aimed to provide a description of the methods we have used at a level that will be appropriate for researchers just venturing into studies of the neural mechanisms of insight and creativity. | Compound Remote Associate problems | Behavior Research |
| 17 | Brunyé et al. (2015) | NeuroReport | General Creative Process | brain function | This study aimed to evaluate the influence of left frontopolar versus auditory (control) cortex transcranial direct current stimulation (tDCS) on the breadth of semantic associations produced in a cued free association task. | Remote Associates Test | Neuroscience Approach |
| 18 | Cai et al. (2009) | Proceedings of the National Academy of Sciences of the United States of America | Insight Problem-solving | incubation effect | This study aimed to examine the role of REM on creative problem solving with the Remote Associates Test (RAT). | Remote Associates Test | Behavior Research |
| 19 | Carlsson et al. (2019) | Creativity Research Journal | General Creative Process | incubation effect | In the present study, daytime napping or resting wakefulness was used to investigate their effects on the Remote Associate Test. Further, it was examined whether a nap would be beneficial for divergent creativity. | Remote Associates Test | Behavior Research |
| 20 | Cerruti and Schlaug (2009) | Journal of Cognitive Neuroscience | General Creative Process | brain function | This study aimed to assess whether modulating excitability at F3 (left dorsolateral prefrontal cortex) could affect complex verbal abilities. | Compound Remote Associate problems | Neuroscience Approach |
| 21 | Chermahini and Hommel (2010) | Cognition | Predictor or Criterion | eye movement | This study aimed to explore whether individual performance in divergent thinking (alternative use task) and convergent thinking (remote association task) can be predicted by the individual spontaneous eye blink rate (EBR). | Dutch version of the RAT | Neuroscience Approach |
| 22 | Chiu and Tu (2014) | Psychological Reports | General Creative Process | priming effect | This study aimed to investigate the service priming effect on creativity performance. | Chinese version of the RAT | Behavior Research |
| 23 | Colzato et al. (2013) | Psychonomic Bulletin and Review | Social Interaction Effect | interpersonal interaction | This study aimed to test whether the degree of self–other integration is systematically affected by the control characteristics of temporally overlapping but unrelated and nonsocial creativity tasks. | Dutch version of the RAT | Behavior Research |
| 24 | Colzato et al. (2017) | Mindfulness | Effect of Treatment | mindfulness | This study aimed to replicate and extend previous findings about attempting to overcome the methodological and theoretical diversity across previous studies by distinguishing between convergent and divergent thinking (RAT and AUT) and between focused-attention (FA) meditation and open-monitoring (OM) meditation, the two main techniques of Buddhist meditation. | Dutch version of the RAT | Behavior Research |
| 25 | Colzato et al. (2018) | Neuropsychologia | General Creative Process | brain function | This study tested a causal relation between vagus nerve and creativity. | Compound Remote Associate problems | Neuroscience Approach |
| 26 | Creswell et al. (2013) | PLoS ONE | Individual Difference | stress | This study aimed to test whether an experimental manipulation of self-affirmation improves problem-solving performance in chronically stressed participants. | Compound Remote Associate problems | Behavior Research |
| 27 | Cristofori et al. (2018) | Cognitive, Affective and Behavioral Neuroscience | Individual Difference | monetary rewards | This study aimed to explore whether the size and/or awareness of reward cues could modulate creative problem solving. | Compound Remote Associate problems | Behavior Research |
| 28 | Cushen and Wiley (2018) | Memory and Cognition | Insight Problem-solving | attentional effect | This study explored the role of individual differences in attentional control and the ability to make remote associations and their possible combined effects on spontaneous analogical transfer. | Compound Remote Associate problems | Behavior Research |
| 29 | Davelaar (2015) | Topics in Cognitive Science | Remote Association | memory effect | This study aimed to evaluate the patch model of foraging in semantic memory on a semantic search task that differs in the number of target items and cues. | Compound Remote Associate problems | Behavior Research |
| 30 | de et al. (2018) | Journal of Creative Behavior | Individual Difference | motivation | This study investigated experimentally whether the relationship between sEBR and divergent and convergent thinking depends on individual differences in affect and motivation. | Dutch version of the RAT | Neuroscience Approach |
| 31 | Denney et al. (2011) | Archives of Clinical Neuropsychology | Clinical Case | other disorders | This study aimed to examine information processing speed in MS patients and controls with a computerized battery of covertly timed as well as explicitly timed measures. | Computerized version of the RAT | Behavior Research |
| 32 | Dewhurst et al. (2011) | Personality and Individual Differences | General Creative Process | memory effect | This study aimed to investigate the relationship between creativity and susceptibility to associative memory illusions in the Deese/Roediger–McDermott procedure. | Compound Remote Associate problems | Behavior Research |
| 33 | Di et al. (2018) | Proceedings of the National Academy of Sciences of the United States of America | Remote Association | brain wave | This study aimed to investigate how alpha oscillations contribute to both convergent and divergent creative cognition and provided a neural mechanism linking these two distinct cognitive processes. | Compound Remote Associate problems | Neuroscience Approach |
| 34 | Ding et al. (2015) | Social Cognitive and Affective Neuroscience | Effect of Treatment | medicine effect | This study aimed to examine how meditation in comparison with relaxation influences the reaction of the participant to a correct solution. | Chinese version of the RAT | Neuroscience Approach |
| 35 | Dong (2018) | NeuroQuantology | General Creative Process | brain structure | This paper studied innovative brain neural mechanisms based on cognitive science. | Remote Associates Test | Neuroscience Approach |
| 36 | Du et al. (2017) | Journal of Creative Behavior | Insight Problem-solving | aha experience | This study aimed to examine whether people can recollect their “Aha!” experiences as well as “non-Aha” ones. | Chinese version of the RAT | Behavior Research |
| 37 | Duan et al. (2019) | Thinking and Reasoning | Individual Difference | stress | This study examined the effects of acute stress on creative problem-solving and reexamines the issue of how stress may affect convergent and divergent problem-solving differentially. | Chinese version of the RAT | Behavior Research |
| 38 | Duan et al. (2019) | Frontiers in Psychology | Individual Difference | stress | The present study examined the relationships among trait anxiety, acute stress, and inhibitory control using a version of the flanker task. | Chinese version of the RAT | Behavior Research |
| 39 | Ellis and Brewer (2018) | Consciousness and Cognition | Insight Problem-solving | memory effect | This study aimed to replicate and further elucidate the relation between WMC and CRAT performance. | Compound Remote Associate problems | Behavior Research |
| 40 | Erickson et al. (2018) | Neuropsychologia | Insight Problem-solving | brain structure | This study aimed to test the hypothesis that trait-like components of resting-state brain activity can predict, and may underlie, qualitative, trait-like individual differences in the tendency to solve problems by insight or analysis, thus signifying an insight-analysis dimension of cognitive style. | Compound Remote Associate problems | Neuroscience Approach |
| 41 | Eskine et al. (2018) | Psychology of Music | Effect of Treatment | music | The goal of the current study was to test whether creativity is greater after listening to music than to non-musical control sounds. | Remote Associates Test | Behavior Research |
| 42 | Faust-Socher et al. (2014) | Annals of Neurology | Clinical Case | Parkinson’s Disease | This study aimed to examine Parkinson disease patients’ ability to perform creativity tasks compared to healthy controls and to verify whether creativity is related to an impulse control disorder (ICD) as a complication of dopaminergic therapy. | Hebrew version of the RAT | Behavior Research |
| 43 | Ferraro and III (2015) | Ecopsychology | Effect of Treatment | outdoors activity | This study aimed to test convergent creativity in undergraduate students following a 6-day wilderness trip to the Boundary Waters Canoe Area Wilderness (BWCAW). | Remote Associates Test | Behavior Research |
| 44 | Fleck and Braun (2015) | Journal of Cognitive Psychology | Effect of Treatment | eye movement | This study aimed to explore whether the bilateral EM effect could be extended to a creativity task that draws heavily on convergent thinking. | Compound Remote Associate problems | Neuroscience Approach |
| 45 | Garner and Howe (2014) | Memory | Insight Problem-solving | memory effect | This study aimed to test the effectiveness of priming from false memories created by incidentally processing information for its survival relevance. | Compound Remote Associate problems | Behavior Research |
| 46 | Godwin et al. (2017) | Neuropsychologia | General Creative Process | brain connectome | This study aimed to examine the extent to which the DMN, along with the dorsal attention network (DAN) and frontoparietal control network (FPCN), correlate with the tendency for the mind to wander in daily life. | Compound Remote Associate problems | Neuroscience Approach |
| 47 | Gold et al. (2012) | Laterality | Effect of Treatment | lateralization | This study aimed to directly examine the relation between verbal creativity and right hemisphere involvement during novel metaphor comprehension. | Hebrew version of the RAT | Behavior Research |
| 48 | Goldstein et al. (2010) | Psychonomic Bulletin and Review | Effect of Treatment | lateralization | This study aimed to explore the possibility of enhancing creative problem solving by artificially activating the RH ahead of time using unilateral hand contractions. | Hebrew version of the RAT | Neuroscience Approach |
| 49 | Gómez-Ariza et al. (2017) | Journal of Experimental Psychology: Learning Memory and Cognition | General Creative Process | memory effect | This study aimed to explore to what extent retrieval-induced forgetting might hinder performance on a subsequent unrelated task that required participants to solve creative verbal problems. | Spanish version of the RAT | Behavior Research |
| 50 | Gupta et al. (2012) | Psychological Science | Test Development | test modeling | This study aimed to investigate individual differences in creativity as measured with a complex problem-solving task by developing a computational model of the remote associates test (RAT). | Compound Remote Associate problems | Behavior Research |
| 51 | Han et al. (2018) | PeerJ | General Creative Process | gene | The present study investigated the genetic basis for both the divergent and the convergent thinking components of creativity. | Chinese version of the RAT | Neuroscience Approach |
| 52 | Harkins (2006) | Journal of Personality and Social Psychology | Predictor or Criterion | self-evaluation | This study aimed to identify the mediating process(es) of the evaluation-performance relationship by performing a molecular analysis of performance on the RAT. | Remote Associates Test | Behavior Research |
| 53 | Hertenstein et al. (2019) | Brain Stimulation | General Creative Process | brain wave | The aim of the present research was to further elucidate the neural basis of and ways to improve creativity based on well-defined behavioral tasks and systematic activity changes of the right and left frontal cortex through tDCS along with electroencephalographic (EEG) monitoring of induced brain activity changes in healthy humans. | Compound Remote Associate problems | Neuroscience Approach |
| 54 | Hong et al. (2019) | Computer Assisted Language Learning | Effect of Treatment | digital learning | This study examined the cognitive and affective factors (intrinsic cognitive load, gameplay interest, and flow experience) affect players’ engagement with the game during game play to gain a better understanding of the impact of games and to more effectively design games for cognitive and affective learning. | Chinese version of the RAT | Behavior Research |
| 55 | Hoşgören et al. (2019) | Noropsikiyatri Arsivi | Clinical Case | bipolar disorder | This study aimed to examine prefrontal cortex (PFC) activity with functional near-infrared spectroscopy (fNIRS) during the RAT and AuT tests in subjects with bipolar disorder (BD). | Turkish version of the RAT | Neuroscience Approach |
| 56 | Howe and Garner (2018) | Memory | Insight Problem-solving | memory effect | This study aimed to investigate whether false memories could be used to bias ambiguous insight-based problem-solving tasks in a similar manner (by increasing solution rates and decreasing solution times.) | Compound Remote Associate problems | Behavior Research |
| 57 | Howe et al. (2010) | Cognition | Insight Problem-solving | memory effect | This study aimed to examine whether false memories can also be used to prime higher order cognitive processes, namely, insight-based problem solving. | Compound Remote Associate problems | Behavior Research |
| 58 | Howe et al. (2011) | Journal of Experimental Child Psychology | Insight Problem-solving | memory effect | This study aimed to investigate whether false memories could prime insight problem-solving tasks. | Compound Remote Associate problems | Behavior Research |
| 59 | Howe et al. (2016) | Memory | Insight Problem-solving | memory effect | This study aimed to clarify the locus of priming effects whereby memory illusions can successfully prime performance on insight-based problems. | Compound Remote Associate problems | Behavior Research |
| 60 | Huang (2017) | Thinking Skills and Creativity | Insight Problem-solving | eye movement | This study aimed to record eye movements while participants solved twelve remote associates problems compiled by Huang (2014). | Chinese version of the RAT | Neuroscience Approach |
| 61 | Huang (2019) | Universal Access in the Information Society | Effect of Treatment | digital learning | 1. Designing a set of teaching schemes and incorporating it into experiential learning. 2. Developing a RAT information system to record question generation and question solving. 3. Exploring the impact of learning activities and the RAT learning system on learners, including RAT performance (question-solving score), associative self-efficacy, perceived importance of teamwork, and perceived usefulness. | Chinese version of the RAT | Behavior Research |
| 62 | Huang et al. (2019) | Thinking Skills and Creativity | Insight Problem-solving | eye movement | The main purpose of this study is to examine the applicability of Representational Change Theory (an insight theory) for remote associates problem-solving. | Chinese version of the RAT | Neuroscience Approach |
| 63 | Hung et al. (2016) | Creativity Research Journal | Test Development | item features | This study aimed to elucidate the dimensionality and the relationship between item features and item difficulties for the RAT—Chinese Version (RAT-C) using the Rasch model and the linear logistic test model (LLTM). | Chinese version of the RAT | Behavior Research |
| 64 | Hutten et al. (2019) | European Neuropsychopharmacology | Effect of Treatment | medicine effect | The present study was set up to test the acute effects of cocaine on objective and self-rated creative performance and to test whether potential behavioral drug effects are associated with personal factors like mood state and trait empathy. | Dutch version of the RAT | Behavior Research |
| 65 | Isen et al. (2004) | Motivation and Emotion | Individual Difference | mood | This study aimed to investigate whether tasting a pleasant product can induce positive affect similar to that induced through other established affect inductions. | Remote Associates Test | Behavior Research |
| 66 | Japardi et al. (2018) | Neuropsychologia | Individual Difference | creative difference | This study aimed to examine the neural substrates underlying divergent and convergent thinking in highly creative individuals relative to a healthy comparison group using AUT and RAT. | Remote Associates Test | Neuroscience Approach |
| 67 | Jarosz et al. (2012) | Consciousness and Cognition | Effect of Treatment | alcohol | This study aimed to test the effects of moderate alcohol intoxication on a common creative problem-solving task (RAT). | Compound Remote Associate problems | Behavior Research |
| 68 | Ji et al. (2018) | Gaojishu Tongxin/Chinese High Technology Letters | Insight Problem-solving | brain connectome | Using the EEG traceability method, the EEG signals recorded by the scalp were used to reconstruct the distribution pattern of the cerebral cortex source signals. The brain cortex source signals were used to construct the epiphany brain network and calculate the attribute parameters of the network to explore the neural mechanism of epiphany. | Chinese version of the RAT | Neuroscience Approach |
| 69 | Jones and Estes (2015) | Thinking and Reasoning | General Creative Process | analogy | This study aimed to investigate the relative influences of divergent and convergent thinking as predictors of verbal analogy performance. | Compound Remote Associate problems | Behavior Research |
| 70 | Kajić et al. (2017) | Frontiers in Psychology | Remote Association | memory effect | This study aimed to present a model (Neural Engineering Framework) that can solve RATs. | Remote Associates Test | Behavior Research |
| 71 | Kaufman et al. (2013) | Personality and Individual Differences | Individual Difference | personality | This study aimed to investigate individual differences in these two constructs and see how personality and creativity are related by major. | Compound Remote Associate problems | Behavior Research |
| 72 | Kazén et al. (2015) | Journal of Personality | Individual Difference | affect regulation | This study aimed to show that (a) differences in affect regulation abilities (“action orientation”) and (b) implicit more so than self-reported affect assessments need to be considered to advance our understanding of these processes. | German version of the RAT | Behavior Research |
| 73 | Kim (2015) | Creativity Research Journal | Effect of Treatment | mindfulness | This study aimed to test whether squeezing a soft versus a hard ball facilitates different types of creativity. | Remote Associates Test | Behavior Research |
| 74 | Kizilirmak et al. (2016) | Frontiers in Psychology | Insight Problem-solving | brain function | This study aimed to investigate induced insight and the successful episodic encoding of insight solutions using a version of the CRAT. | German version of the RAT | Neuroscience Approach |
| 75 | Kizilirmak et al. (2016) | Journal of Problem Solving | Insight Problem-solving | memory effect | This study aimed to examine two kinds of memory measures: indirect (solving old and new problems on a test) and direct (recognition memory). | German version of the RAT | Behavior Research |
| 76 | Kizilirmak et al. (2018) | Frontiers in Psychology | Insight Problem-solving | intuition | This study aimed to investigate the subjective feeling of closeness to the solution, assessed as feeling-of-warmth (FoW), its relationship to solving the problem versus being presented with it, and whether a feeling of Aha! was experienced. | Compound Remote Associate problems | Behavior Research |
| 77 | Klein and Badia (2015) | Journal of Creative Behavior | Remote Association | memory effect | This study aimed to explore how complex creative relations can arise from fairly frequent semantic relations observed in everyday language. | Remote Associates Test | Behavior Research |
| 78 | Knott et al. (2014) | Journal of Cognitive Psychology | Individual Difference | affect regulation | This study aimed to investigate the effect of emotional mood states on the ability to create effective primes using the recently developed false memory priming paradigm. | Compound Remote Associate problems | Behavior Research |
| 79 | Kohn and Smith (2009) | Journal of Creative Behavior | Insight Problem-solving | incubation effect | This study aimed to examine the effects of incubation on initially unsolved Remote Associates Test (RAT) problems. | Remote Associates Test | Behavior Research |
| 80 | Koppel and Storm (2014) | Memory | General Creative Process | incubation effect | This study aimed to test the hypothesis that retrieval-induced forgetting would not correlate with RAT performance during the post-incubation block of the distributed condition. | Remote Associates Test | Behavior Research |
| 81 | Kowal et al. (2015) | Psychopharmacology | Clinical Case | brain injury | This study aimed to investigate the acute effects of cannabis on creativity. | Dutch version of the RAT | Behavior Research |
| 82 | Kraus and Holtgraves (2018) | Journal of Creative Behavior | Insight Problem-solving | aha experience | The purpose of this experiment was to examine semantic priming in the RH for CRA solution words after attempting to solve CRA problems. | Compound Remote Associate problems | Behavior Research |
| 83 | Kuhl and Kazén (2008) | Journal of Personality and Social Psychology | Effect of Treatment | lateralization | This study aimed to examine to what extent information related to different social needs is associated with hemispheric laterality. | Compound Remote Associate problems | Behavior Research |
| 84 | LeBoutillier and Barry (2018) | Creativity Research Journal | Predictor or Criterion | psychological mindedness | This study aimed to investigate the role of two PM measures and personality in predicting creative cognition performance. | Remote Associates Test | Behavior Research |
| 85 | Lee et al. (2014) | Psychology of Aesthetics, Creativity, and the Arts | Test Development | item features | This study aimed to examine the internal and external structure validity evidence of a computer-based, 30-item RAT based on scores from a sample of undergraduate students. | Compound Remote Associate problems | Behavior Research |
| 86 | Li et al. (2019) | Frontiers in Psychology | General Creative Process | brain structure | In the present study, the relationship between gray matter density (GMD)/white matter density (WMD) and RAT was explored using voxel-based morphometry (VBM). | Chinese version of the RAT | Neuroscience Approach |
| 87 | Marko (2016) | Journal of Psychology: Interdisciplinary and Applied | Individual Difference | stress | This study aimed to provide necessary evidence to support the protocol validity (i.e., to support its ability to induce a stress response) at both the physiological and psychological levels. | Remote Associates Test | Behavior Research |
| 88 | Marko et al. (2018) | Behavior Research Methods | Test Development | item features | This study aimed to evaluate the heterogeneity and remoteness of RAT problems using both score- and item-level analyses and the involvement of lexical-semantic and executive functioning in RAT. | Slovak version of the RAT | Behavior Research |
| 89 | Martinsen (2011) | Creativity Research Journal | Individual Difference | personality | This study aimed to investigate the structure and the predictive power of a large number of creativity-relevant constructs. | Norwegian version of the RAT | Behavior Research |
| 90 | Martinsen and Furnham (2015) | Learning and Individual Differences | Individual Difference | motivation | This study aimed to explore the conditional relationship between the Assimilator–Explorer (A–E) cognitive styles and performance on complex, structured tasks. | Compound Remote Associate problems | Behavior Research |
| 91 | Mikulincer and Sheffi (2000) | Motivation and Emotion | Individual Difference | mood | This study aimed to examine the moderating effect of attachment style on cognitive reactions to positive affect inductions. | Remote Associates Test | Behavior Research |
| 92 | Mohamed (2016) | Journal of Creative Behavior | Effect of Treatment | medicine effect | This study aimed to investigate the effects of modafinil on reliable measures of divergent and convergent thinking tasks of creativity in healthy volunteers. | Remote Associates Test | Behavior Research |
| 93 | Morrison et al. (2017) | Journal of Creative Behavior | Insight Problem-solving | incubation effect | This study aimed to explore whether taking time away from a problem (i.e., incubation) may serve to change the nature of neurocognitive processing occurring during incubation, as indexed by the experience of insight. | Compound Remote Associate problems | Behavior Research |
| 94 | Moss et al. (2011) | Journal of Experimental Psychology: Learning Memory and Cognition | General Creative Process | priming effect | This study aimed to examine how the time at which problem solving is suspended relative to an impasse affects the impact of incidental hints. | Compound Remote Associate problems | Behavior Research |
| 95 | Mussel et al. (2015) | European Journal of Personality | General Creative Process | intellengence | This study aimed to investigate how the construct intellect, according to the Theoretical Intellect Framework (TIF), predicts creativity. | Compound Remote Associate problems | Behavior Research |
| 96 | Nagaya and Nakayachi (2017) | Shinrigaku Kenkyu | Predictor or Criterion | regulatory focus theory | This study aimed to investigate the hypothesis that prevention-focused individuals employ a persistent style when performing creative tasks, considering two kinds of regulatory focus (chronic/situational). | Japanese version of the RAT | Behavior Research |
| 97 | Nam and Lee (2015) | Psychologia | Insight Problem-solving | incubation effect | This study aimed to investigate an unconscious aspect of immediate incubation effect in creativity. | Korean version of the RAT | Behavior Research |
| 98 | Ogawa et al. (2018) | Scientific Reports | Insight Problem-solving | brain wave | This study aimed to investigate large-scale networks associated with insight problem solving. | Japanese version of the RAT | Neuroscience Approach |
| 99 | Olteţeanu and Falomir (2015) | Pattern Recognition Letters | Test Development | test modeling | This study aimed to explore (a) whether a convergence process can be used to solve such queries and (b) if frequency of appearance of the test items in language data may influence knowledge association or discovery in solving such problems. | Computerized version of the RAT | Modeling |
| 100 | Olteteanu and Schultheis (2017) | Journal of Creative Behavior | Test Development | item features | This study aimed to explore the nature of the associative process by manipulating (a) the frequency with which a pair of items are associated as associative strength, and (b) the probability of reaching the answer given the strength and the spread. | Compound Remote Associate problems | Behavior Research |
| 101 | Olteţeanu et al. (2018) | Behavior Research Methods | Test Development | test modeling | This study aims to enrich the existing pool of compound Remote Associates Test items and provide a standardized treatment which allows control over the frequency of occurrence and probability of finding an answer. | Computerized version of the RAT | Modeling |
| 102 | Olteţeanu et al. (2019) | Knowledge-Based Systems | Test Development | test modeling | This study aimed to use the two points put forward by Worthen and Clark creatively, setting to computationally construct a set of functional RAT items using a modern set of word association norms. | Computerized version of the RAT; Compound Remote Associate problems | Modeling |
| 103 | Olteţeanu et al. (2019) | Frontiers in Psychology | Test Development | new version development | This paper focuses on the construction and norming of a variant of the RAT in Romanian. | English version of the Compound Remote Associate problems; Italian version of the RAT; Romanian version of the RAT | Behavior Research |
| 104 | Op et al. (2018) | European Journal of Work and Organizational Psychology | Predictor or Criterion | job performance | This study aimed to build on the proactivity and energy-at-work literatures to argue that individuals may proactively manage their levels of physical and mental energy to promote their own work. | Dutch version of the RAT | Behavior Research |
| 105 | Orita et al. (2018) | Shinrigaku Kenkyu | Test Development | new version development | This paper aimed to propose a new Japanese version of the RAT, devising a set of 80 RAT problems that were intended to have the solver reach an impasse by evoking a certain fixed term. | Japanese version of the RAT | Behavior Research |
| 106 | Peña et al. (2019) | Scientific Reports | General Creative Process | brain function | This study was to investigate the effect of tRNS on both verbal convergent and (verbal and visual) divergent thinking during left DLPFC tRNS stimulation. | Spanish version of the RAT | Neuroscience Approach |
| 107 | Penaloza and Calvillo (2012) | Creativity Research Journal | Insight Problem-solving | incubation effect | This study aimed to test the forgetting-fixation account with RATs, using a trial-by-trail method, and isolating a reading task as the incubation task. | Remote Associates Test | Behavior Research |
| 108 | Pick and Lavidor (2019) | Neuropsychologia | Remote Association | brain function | This study aimed to explore the sub-components of the RAT, and to link them to angular gyrus (AG) activation. | Hebrew version of the RAT | Neuroscience Approach |
| 109 | Polner et al. (2018) | PeerJ | Clinical Case | schizophrenia | This study aimed to test the hypothesis that positive, impulsive, and disorganized schizotypy will demonstrate stronger associations with indicators of creativity, if the effect of mental health, insomnia, and intellect are statistically controlled. | Hungarian version of the RAT | Behavior Research |
| 110 | Radel et al. (2015) | Cognition | General Creative Process | priming effect | This study aimed to pursue the possibility that the central mechanism associated with this effect might be a reduced capacity to exert inhibition. | French version of the RAT | Behavior Research |
| 111 | Razumnikova (2007) | Brain Research Bulletin | General Creative Process | brain wave | This study aimed to reveal how anterior and posterior cortical regions of two hemispheres may interact during the formation of new original verbal associations, and how different neural oscillations may be included in creativity-related cooperation of cortical networks. | Russian version of the RAT | Neuroscience Approach |
| 112 | Razumnikova and Bryzgalov (2006) | Neuroscience and Behavioral Physiology | Individual Difference | gender difference | This study aimed to explore gender-related differences in the EEG correlates of creative thought by mapping EEG power during performance of a RAT as compared with verbal word-generation and simple association tasks. | Computerized version of the RAT | Neuroscience Approach |
| 113 | Renner and Beversdorf (2010) | Neurocase | Individual Difference | stress | This study aimed to examine the effects of psychological stress on cognitive flexibility in problem solving and immediate memory with a more naturalistic psychological stressor, and the interaction between subject ability and the cognitive effects of psychological stress. | Compound Remote Associate problems | Behavior Research |
| 114 | Ricks et al. (2007) | Memory and Cognition | General Creative Process | memory effect | This study aimed to examine how working memory capacity (WMC) relates to performance on a Remote Associates Task (RAT). | Remote Associates Test | Behavior Research |
| 115 | Rigon et al. (2018) | Brain Injury | Clinical Case | other disorders | This study aimed to determine whether convergent problem solving, which contributes to creative thinking, is impaired following TBI. | Compound Remote Associate problems | Behavior Research |
| 116 | Rook (2014) | Creativity Research Journal | Individual Difference | motivation | This study aimed to explore the notion that the effect of the color red on creative thinking varies depending on someone’s appetitive (vs. aversive) motivational orientation prior to the creative act. | Remote Associates Test | Behavior Research |
| 117 | Rothmaler et al. (2017) | Neuropsychologia | Insight Problem-solving | brain wave | This study aimed to investigate the differences and similarities between intrinsic and extrinsic insight on the behavioral as well as neurophysiological level. | German version of the RAT | Neuroscience Approach |
| 118 | Rowe et al. (2007) | Proceedings of the National Academy of Sciences of the United States of America | Individual Difference | mood | This study aimed to examine the thesis that positive affect may serve to broaden the scope of attentional filters, reducing their selectivity. | Remote Associates Test | Behavior Research |
| 119 | Ruggiero et al. (2018) | Creativity Research Journal | Insight Problem-solving | brain function | This study aimed to investigate whether convergent and divergent thinking, as well as insight, can be modulated by transcranial direct current stimulation (tDCS) over the anterior temporal lobe (ATL). | Remote Associates Test | Neuroscience Approach |
| 120 | Salvi et al. (2016) | Quarterly Journal of Experimental Psychology | Individual Difference | cognitive style | This study aimed to investigate if, and how, political orientation is related to the way people solve problems. | Compound Remote Associate problems | Behavior Research |
| 121 | Salvi et al. (2016) | Behavior Research Methods | Test Development | new version development | This study aimed to expand the study of insight problem solving to the Italian language and culture by creating and testing an Italian version of the CRA problems and rebus puzzles. | Italian version of the RAT | Behavior Research |
| 122 | Sandkühler and Bhattacharya (2008) | PLoS ONE | Insight Problem-solving | brain wave | This study aimed to investigate the neural mechanisms of salient features of insightful problem solving in a unified framework. | Compound Remote Associate problems | Neuroscience Approach |
| 123 | Sassenberg et al. (2017) | Journal of Experimental Social Psychology | Remote Association | priming effect | This study aimed to test the hypothesis that implicitly priming creativity results in more creativity (i.e., flexibility). | German version of the RAT | Behavior Research |
| 124 | Schmajuk et al. (2009) | Creativity Research Journal | General Creative Process | attentional effect | This article aimed to introduce an attentional–associative model of classical conditioning that can be applied to the description of the mechanisms associated with creative processes. | Remote Associates Test | Behavior Research |
| 125 | Schuler et al. (2019) | NeuroImage | General Creative Process | brain connectome | This study employed functional connectivity analyses of resting-state functional magnetic imaging data in order to shed light on these neural underpinnings of creative aspects so as to draw a comprehensive picture of personality traits associated with the brain structures of the dopaminergic system involved in insight and creativity. | German version of the RAT | Neuroscience Approach |
| 126 | Schwartz and Canetti (2014) | Rorschachiana | Individual Difference | mood | This study aimed to contribute to the understanding of how creativity can be manifested in Rorschach scores and to investigate the train of research showing a connection between emotional distress and creativity in light of the Rorschach measures. | Hebrew version of the RAT | Behavior Research |
| 127 | Shen et al. (2016) | International Journal of Psychophysiology | Insight Problem-solving | brain function | This study aimed to determine whether domain-generality theory or domain-specificity theory more accurately describes insight. | Compound Remote Associate problems | Neuroscience Approach |
| 128 | Shen et al. (2016) | American Journal of Psychology | Test Development | new version development | This study aimed to develop a Chinese version of the CRA test with a sufficient number of items based on Mednick’s (1962) associative theory. | Chinese version of the RAT | Behavior Research |
| 129 | Shen et al. (2018) | Creativity Research Journal | Predictor or Criterion | risk-taking | This study aimed to systemically investigate whether risk-taking is linked to convergent thinking. | Chinese version of the RAT | Behavior Research |
| 130 | Simor and Polner (2017) | Chronobiology International | Individual Difference | sleep | This study aimed to examine convergent and divergent thinking abilities in late and early chorotypes, taking into consideration the influence of asynchrony (optimal versus non-optimal testing times) and sleep quality. | Hungarian version of the RAT | Behavior Research |
| 131 | Sio and Ormerod (2015) | Thinking and Reasoning | Insight Problem-solving | incubation effect | This study aimed to examine the link between spreading activation and cue assimilation in solving a class of verbal insight problems, and to extend the generality of known incubation effects. | Compound Remote Associate problems | Behavior Research |
| 132 | Sio and Rudowicz (2007) | Creativity Research Journal | Insight Problem-solving | incubation effect | This study aimed to test the spreading-activation hypothesis that an incubation period helps to sensitize problem solvers to relevant concepts, and the selective forgetting hypothesis that an incubation period helps to desensitize problem solvers to irrelevant concepts. | Compound Remote Associate problems | Behavior Research |
| 133 | Sio et al. (2013) | Memory and Cognition | General Creative Process | incubation effect | This study aimed to compare the effect of sleep on different RAT problems varying in terms of problem difficulty. | Compound Remote Associate problems | Behavior Research |
| 134 | Sio et al. (2017) | Memory and Cognition | Insight Problem-solving | incubation effect | This study examined whether distributed effort can provide an advantage in problem solving, particularly for problems that can induce fixation, and whether and how incubation can be combined with distributed effort to further enhance performance. | Compound Remote Associate problems | Behavior Research |
| 135 | Smith et al. (2013) | Cognition | General Creative Process | incubation effect | This study aimed to evaluate how people generated these guesses by using Latent Semantic Analysis to measure the similarity between the guesses, cues, and answers. | Compound Remote Associate problems | Behavior Research |
| 136 | Storm and Hickman (2015) | Quarterly Journal of Experimental Psychology | Insight Problem-solving | metacognition | This study aimed to investigate the influence of exposure to fixating information on predictions of problem-solving performance. | Remote Associates Test | Behavior Research |
| 137 | Storm et al. (2011) | Journal of Experimental Psychology: Learning Memory and Cognition | General Creative Process | memory effect | This study aimed to examine whether an analogous phenomenon occurs in the context of creative problem solving. | Remote Associates Test | Behavior Research |
| 138 | Strick et al. (2012) | Consciousness and Cognition | Effect of Treatment | mindfulness | This study aimed to test the hypothesis that Zen meditation increases access to accessible but unconscious information. | Remote Associates Test | Behavior Research |
| 139 | Suzuki and Usher (2009) | Personality and Individual Differences | Clinical Case | schizophrenia | This study aimed to test the hypothesis that the level of reduced laterality in language is correlated with the degrees of schizotypal personality in healthy individuals and with their performance on the remote associates task. | Compound Remote Associate problems | Behavior Research |
| 140 | Tempest and Radel (2019) | Behavioural Brain Research | Insight Problem-solving | brain function | This study employed a verb generation task to (i) independently investigate creativity using a modified verb generation task (cued vs. uncued conditions) administered in French, (ii) assess the validity of task responses with additional measures of creativity, and (iii) determine the utility of functional near infrared spectroscopy (fNIRS) to measure changes in the frontopolar cortex during augmented states of creativity. | Compound Remote Associate problems | Neuroscience Approach |
| 141 | Terai et al. (2013) | Shinrigaku kenkyu : The Japanese journal of psychology | Test Development | new version development | This study aimed to provide a brief overview of the Japanese version of RAT that had been developed. | Japanese version of the RAT | Behavior Research |
| 142 | Thompson (2004) | British Journal of Educational Psychology | Individual Difference | personality | This study aimed to seek an explanation of the differing effects of these forms of performance feedback, testing the assumption that students high in self-handicapping behavior would react more negatively following noncontingent success. | Remote Associates Test | Behavior Research |
| 143 | Thompson and Dinnel (2007) | Educational Psychology | Individual Difference | self-worth | This study aimed to assess the veracity of two understandings of self-worth protection, which are (1) poor performance of students high in self-worth protection in situations of high evaluative threat is viewed as self-handicapping behaviors, and (2) poor performance of students high in self-worth protection is an outcome of anxiety. | Remote Associates Test | Behavior Research |
| 144 | Thompson and Dinnel (2007) | Educational Psychology | Individual Difference | self-worth | This study aimed to test the viability of the self-worth account of poor performance where it might be least likely to be seen, in undergraduate women in mathematics, where a helplessness account and the negative effects of stereotype threat have been proposed. | Remote Associates Test | Behavior Research |
| 145 | Threadgold et al. (2019) | Applied Cognitive Psychology | Effect of Treatment | music | This study examine the claim that background music enhances creativity by employing variants of widely used verbal problem-solving tasks that are typically used to study creativity being indexed by, and solved via, a process of insight: Compound Remote Associate Tasks. | Compound Remote Associate problems | Behavior Research |
| 146 | Tik et al. (2018) | Human Brain Mapping | Insight Problem-solving | brain function | This study aimed to investigate insightful problem-solving using fMRI for the first time in an ultra-high magnetic field. | German version of the RAT | Neuroscience Approach |
| 147 | Toivainen et al. (2019) | Frontiers in Psychology | Test Development | new version development | The present study investigated the relationship between the linguistic and a newly developed visual version of RAT in Russian and Finnish native speakers. | Compound Remote Associate problems; visual version of ther RAT; linguistic version of ther RAT; functional version of ther RAT | Behavior Research |
| 148 | Topolinski and Deutsch (2012) | Experimental Psychology | Individual Difference | affect regulation | This study aimed to demonstrate phasic affective modulation of creativity and describe the mechanisms. | German version of the RAT | Behavior Research |
| 149 | Tu et al. (2017) | Journal of Affective Disorders | Clinical Case | bipolar disorder | This study aimed to investigate the structural correlates of creative thinking in patients with bipolar disorder (BD) to understand the possible neural mechanism of creative thinking in BD. | Chinese version of the RAT | Neuroscience Approach |
| 150 | Turner et al. (2017) | Laterality | Effect of Treatment | lateralization | This study aimed to assess the reliability and generality of the RAT and line bisection findings following unilateral hand contractions. | Compound Remote Associate problems | Behavior Research |
| 151 | Ueda et al. (2016) | Psychological Research | Predictor or Criterion | eye movement | This study aimed to provide behavioral evidence concerning relationships between spontaneous eye blinks (during the AUT, RAT, and at rest) and creative performance. | Japanese version of the RAT | Neuroscience Approach |
| 152 | van et al. (2011) | Personality and Social Psychology Bulletin | Individual Difference | motivation | This study aimed to test the hypothesis that the emotion of benign envy, but not the emotions of admiration or malicious envy, motivates people to improve themselves. | Remote Associates Test | Behavior Research |
| 153 | Vul and Pashler (2007) | Memory and Cognition | Insight Problem-solving | incubation effect | This study aimed to investigate three potential accounts of incubation in retrieval and search problems (subconscious work, spreading activation, and fixation forgetting). | Compound Remote Associate problems | Behavior Research |
| 154 | Wang et al. (2019) | Cognition | General Creative Process | memory effect | This study tested whether inhibitory control processes underlying retrieval suppression alter the influence of a memory’s underlying semantic content on later thought. | Remote Associates Test | Behavior Research |
| 155 | Ward et al. (2008) | British Journal of Psychology | Individual Difference | cognitive style | This study aimed to investigate the creativity of synesthetes with two measures, AUT and RAT. | Remote Associates Test | Behavior Research |
| 156 | Weinstein et al. (2010) | Personality and Social Psychology Bulletin | Social Interaction Effect | interpersonal interaction | This study aimed to examine interaction quality and joint performance on two creative tasks in unacquainted dyads primed for autonomy or control orientations. | Remote Associates Test | Behavior Research |
| 157 | White and Shah (2006) | Personality and Individual Differences | Clinical Case | ADHD | This study aimed to compare adults with and without ADHD on convergent thinking, divergent thinking, and inhibitory control tasks. | Remote Associates Test | Behavior Research |
| 158 | Whitehurst et al. (2016) | Proceedings of the National Academy of Sciences of the United States of America | General Creative Process | incubation effect | This study aimed to investigate the contribution of the ANS for sleep-dependent memory consolidation by examining post-nap performance changes on the Remote Associates Test (RAT). | Remote Associates Test | Neuroscience Approach |
| 159 | Wronska et al. (2018) | Frontiers in Psychology | General Creative Process | attentional effect | This study tested the idea that engaging in creative activity leads to a broader attentional field. | Polish version of the RAT | Behavior Research |
| 160 | Wu (2019) | Thinking Skills and Creativity | Test Development | item features | This study aimed to compare the three CRAT versions and identify their unique features. | Chinese version of the RAT | Behavior Research |
| 161 | Wu and Chen (2017) | Behavior Research Methods | Test Development | new version development | This study aimed to develop Chinese compound remote associate problems and analyze the passing rates by items, problem solving, times, and various normative data. | Chinese version of the RAT | Behavior Research |
| 162 | Wu and Chen (2019) | Creativity Research Journal | Predictor or Criterion | humor | This study explored the relationship of the different dimensions of cognitive creativity and incongruity-resolution and nonsense humor comprehension, and examined the influence of divergent thinking, insight, and remote association on incongruity-resolution and nonsense humor comprehension. | Chinese version of the RAT | Behavior Research |
| 163 | Wu et al. (2016) | PLoS ONE | Remote Association | brain connectome | This study aimed to analyze the brain network structure and the connection efficiency of the nodes between different distance associations. | Chinese version of the RAT | Neuroscience Approach |
| 164 | Wu et al. (2017) | Thinking Skills and Creativity | Test Development | item features | This study aimed to initiate a new approach to redesign the CRAT by developing a set of remote associate items with low-frequency vocabulary and a set of close associate items with high-frequency vocabulary according to the associative hierarchies. | Chinese version of the RAT | Behavior Research |
| 165 | Xia et al. (2016) | Frontiers in Psychology | General Creative Process | color effect | This study aimed to investigate the effect of color on cognitive task performance. | Chinese version of the RAT | Behavior Research |
| 166 | Xiao et al. (2016) | Beijing Daxue Xuebao (Ziran Kexue Ban)/Acta Scientiarum Naturalium Universitatis Pekinensis | Test Development | new version development | This study aimed to construct a Chinese version of the Remote Associates Test (RAT) based on the theory of associative creativity. | Chinese version of the RAT | Behavior Research |
| 167 | Zhong et al. (2008) | Psychological Science | General Creative Process | incubation effect | This study aimed to examine the effect of unconscious thought on two outcomes of a remote-association test (RAT): implicit accessibility and conscious reporting of answers. | Compound Remote Associate problems | Behavior Research |
| 168 | Zhou et al. (2019) | Frontiers in Psychology | Remote Association | brain wave | The current study focused on the neural mechanisms of two different forms of Zhongyong thinking (IT and ET) on creative problem-solving, investigating the effects of the two divergent thinking forms of Zhongyong on performance levels on the Remote Associates Test (RAT). | Chinese version of the RAT | Neuroscience Approach |
| 169 | Zilm et al. (2019) | Health Environments Research and Design Journal | Individual Difference | expertise | This article reviewed research on the personality characteristics of creative architects and presented the findings of a study of contemporary healthcare architects’ personality types with a focus on the factors that influence creativity in healthcare design. | Remote Associates Test | Behavior Research |
| 170 | Zmigrod and Zmigrod (2016) | Multisensory Research | Predictor or Criterion | perception | This study aimed to examine the manner in which high-level problem solving can be understood in terms of its underpinnings in the perceptual system and the way this system binds multisensory information. | Computerized version of the RAT | Behavior Research |
| 171 | Zmigrod et al. (2015) | Frontiers in Psychology | General Creative Process | attentional effect | This study aimed to test the impact of more stable attention-related preferences. | Dutch version of the RAT | Behavior Research |
| 172 | Zmigrod et al. (2019) | Psychology of Aesthetics, Creativity, and the Arts | Insight Problem-solving | attentional effect | This study examine whether the way in which the brain processes task-irrelevant information affects its ability to solve complex and creative problems. | Computerized version of the RAT | Behavior Research |
